# Electronic profiling of neuronal extracellular vesicles enables early prediction of Parkinson’s disease

**DOI:** 10.1126/sciadv.aee4525

**Published:** 2026-09-30

**Authors:** Tianrui Chang, Cheng Jiang, Shijun Yan, Shofarul Wustoni, Luca Salvigni, Rania Almaghrabi, Michele T. Hu, George K. Tofaris, Keying Guo, Sahika Inal

**Affiliations:** ^1^ Organic Bioelectronics Laboratory, Biological and Environmental Science and Engineering Division, King Abdullah University of Science and Technology (KAUST), Thuwal 23955-6900, Saudi Arabia.; ^2^ Nuffield Department of Clinical Neurosciences, University of Oxford, Oxford, UK.; ^3^ Kavli Institute for Nanoscience Discovery, University of Oxford, Oxford, UK.; ^4^ School of Medicine, Life and Health Sciences, Chinese University of Hong Kong, Shenzhen, China.; ^5^ Department of Pathology, the First Affiliated Hospital, Zhejiang University School of Medicine, Hangzhou, Zhejiang, 310003, China.; ^6^ Department of Biotechnology and Food Engineering, Guangdong Technion–Israel Institute of Technology (GTIIT), Guangdong 515063, China.

## Abstract

Misfolded alpha-Synuclein (α-Syn) accumulation is the defining molecular pathology of Parkinson’s disease (PD), yet blood-based assays remain challenged by the high background of peripheral α-Syn originating mainly from red blood cells. Neuronal extracellular vesicles (EVs) circulating in blood offer a biologically enriched source of brain-derived α-Syn proteoforms, but their ultralow abundance has hindered reliable detection. Here, we introduce an organic electrochemical transistor–based multiparametric diagnostic assay that simultaneously detects total, aggregated, and serine-129–phosphorylated α-Syn proteoforms in serum L1CAM^+^ EVs. The platform achieves low femtomolar sensitivity in buffer and robust analytical performance in clinical specimens. In a cohort of 66 individuals, including prodromal and clinically diagnosed patients with PD, combining the three proteoform readouts distinguished PD from controls with 90.9% accuracy (88.6% sensitivity and 90.9% specificity). By electronically amplifying and resolving multiple neuronal α-Syn signatures in blood, this assay enables a minimally invasive diagnostic tool for early-stage PD and provides a path toward therapeutic intervention during the window when disease-modifying treatments are likely to be most effective.

## INTRODUCTION

Parkinson’s disease (PD) is a progressive neurodegenerative disorder characterized pathologically by the presence of Lewy bodies, intracellular inclusions composed primarily of aggregated alpha-Synuclein (α-Syn) (*1*, *2*). The prodromal phase of PD can be more than a decade long, with nonmotor symptoms and subtle motor changes emerging before clinical diagnosis. Idiopathic REM sleep behavior disorder (iRBD) is one of the strongest predictors of synucleinopathy, with up to 80% of patients converting to PD or related disorders within 12 years (*3*). Yet, iRBD is not present in all patients, underscoring the need for objective biomarkers of the prodromal phase.

PD diagnosis currently relies on clinical assessment of cardinal motor features, which is subjective and often fails to capture disease in its preclinical stages. While imaging techniques can aid diagnosis, they are costly, not broadly accessible or suited for population-level screening (*4*). Crucially, no blood-based test can reliably detect PD pathology. Existing assays for total or pathological α-Syn have produced inconsistent results, and the detection methods are suboptimal (*5*–*9*). There is an urgent demand for sensitive and specific diagnostic tools capable of identifying synucleinopathy at its earliest stages, well before irreversible neuronal damage and motor or cognitive impairments emerge, ideally from an accessible biofluid such as blood (*10*, *11*).

Recent mass spectrometry-based proteomic phenotyping has identified a panel of blood-based biomarkers that distinguish PD from healthy controls (HC) up to 7 years before diagnosis, but this approach requires complex sample preprocessing and lacks sensitivity for low-abundance phosphorylated proteins (*12*, *13*). Among the proposed molecular markers, total α-Syn, aggregated α-Syn, and its phosphorylated form at serine-129 (pSer129) hold strong potential for early diagnosis and therapeutic intervention. However, their measurement in blood is complicated by the overwhelming abundance of peripheral α-Syn—approximately 95% of circulating α-Syn arises from red blood cells, and even mild hemolysis obscures disease-relevant signals (*14*).

Neuronal extracellular vesicles (EVs) offer a solution. These nanoscale, membrane-bound particles, released from neurons and present in blood, carry CNS-derived molecular cargo including α-Syn. Recent multicenter studies have demonstrated that quantifying the total α-Syn in L1CAM^+^ neuronal EVs isolated from serum can reliably predict or diagnose PD (*1*, *10*, *15*–*17*). Yet, one key translational challenge persists: the quantification of extremely low concentrations of α-Syn cargo, particularly pSer129. (i) The specific capture of EVs from complex serum and their controllable release and (ii) overcoming these analytical challenges are essential for clinical deployment of EV-derived biomarkers.

The organic electrochemical transistor (OECT) has recently emerged as a powerful amplifying transducer for biosensing, offering superior signal-to-noise ratio compared to the majority of conventional diagnostic techniques while enabling label-free, rapid, and real-time biomarker detection (*18*). Operating in aqueous environments, OECTs use ion-permeable polymeric semiconductors in the channel and gate electrodes that modulate channel conductivity via electrolyte ions. Their high transconductance allows even a few biochemical binding events at the gate electrode to be transduced into large current responses from the channel, providing intrinsic amplification and hence enabling miniaturization, rendering them ideally suited for point-of-care biosensing applications (*19*). OECT-based assays have achieved femtomolar sensitivity for diverse protein targets, including Alzheimer’s disease biomarkers (*20*, *21*) and SARS-CoV-2 antigens in complex biofluids, including human serum and saliva (*22*–*25*). Their compact architecture and modular gate functionalization make them well suited for multiplexed molecular diagnostics.

Here, we present an OECT-based multiplexed immunosensor that enables early prediction of PD pathology through ultrasensitive detection of α-Syn proteoforms in L1CAM^+^ EVs (L1EVs) immunocaptured from clinical samples. Each gate electrode is functionalized with a subtype-specific antibody, enabling parallel measurement of three disease-relevant α-Syn proteoforms within 40 min. We validated the platform in a cohort of 66 participants, including individuals with PD (*n* = 19), iRBD (*n* = 25), and HC (*n* = 22). The multiplexed readouts differentiated PD and iRBD from controls with 86.8% sensitivity and 89.5% specificity, with pSer129 α-Syn showing the strongest discriminative performance. By integrating an EV enrichment protocol with the intrinsic electronic amplification of the organic transistor enabling the detection of disease-relevant proteoforms at ultralow concentrations, this platform demonstrates a mechanistically grounded, minimally invasive approach for early identification of synucleinopathy. The method is readily generalizable to other biomarker panels and disease contexts requiring high sensitivity to low concentrations and multiplexed detection.

## RESULTS

### Device design

To enable multiplex detection of disease-relevant α-Syn species, we designed OECT-based sensors targeting total α-Syn, aggregated α-Syn, and pSer129 α-Syn, along with an internal negative control (Fig. 1A). Total α-Syn includes monomeric and aggregated forms, whereas the aggregated and pSer129 antibodies selectively bind misfolded and pathogenic proteoforms, respectively. The sensor architecture consists of a top-gated OECT configuration in which a gate-electrode array is positioned above an electrolyte layer and aligned with the underlying OECT channel on a glass substrate (Fig. 1B, left). The transistor channel (width, 100 μm; length, 10 μm) was patterned with the solution-processable p-type mixed conductor, p(g_3_C_2_T2-T), using standard photolithography and parylene C peel-off techniques (*20*, *22*). The gold gate electrode controlled the conductivity of the p(g_3_C_2_T_2_-T) channel by injecting anions into it from the electrolyte, i.e., phosphate buffer (PB, 40 mM), producing the characteristic p-type accumulation-mode transistor response. The devices turned on with an increase in source-drain current (*I*_ds_) [measured at the drain voltage (*V*_ds_) scanned from 0 to −0.5 V] when the gate voltage (*V*_gs_) became more negative than −0.16 V (threshold voltage, *V*_th_) (fig. S1, A and B). Under repeated on-off pulsing (*V*_gs_ steps every 5 s for 5000 s), the OECTs maintained ∼100% of their initial current (fig. S1C), confirming their stability under prolonged electrical operation.

**Fig. 1.**
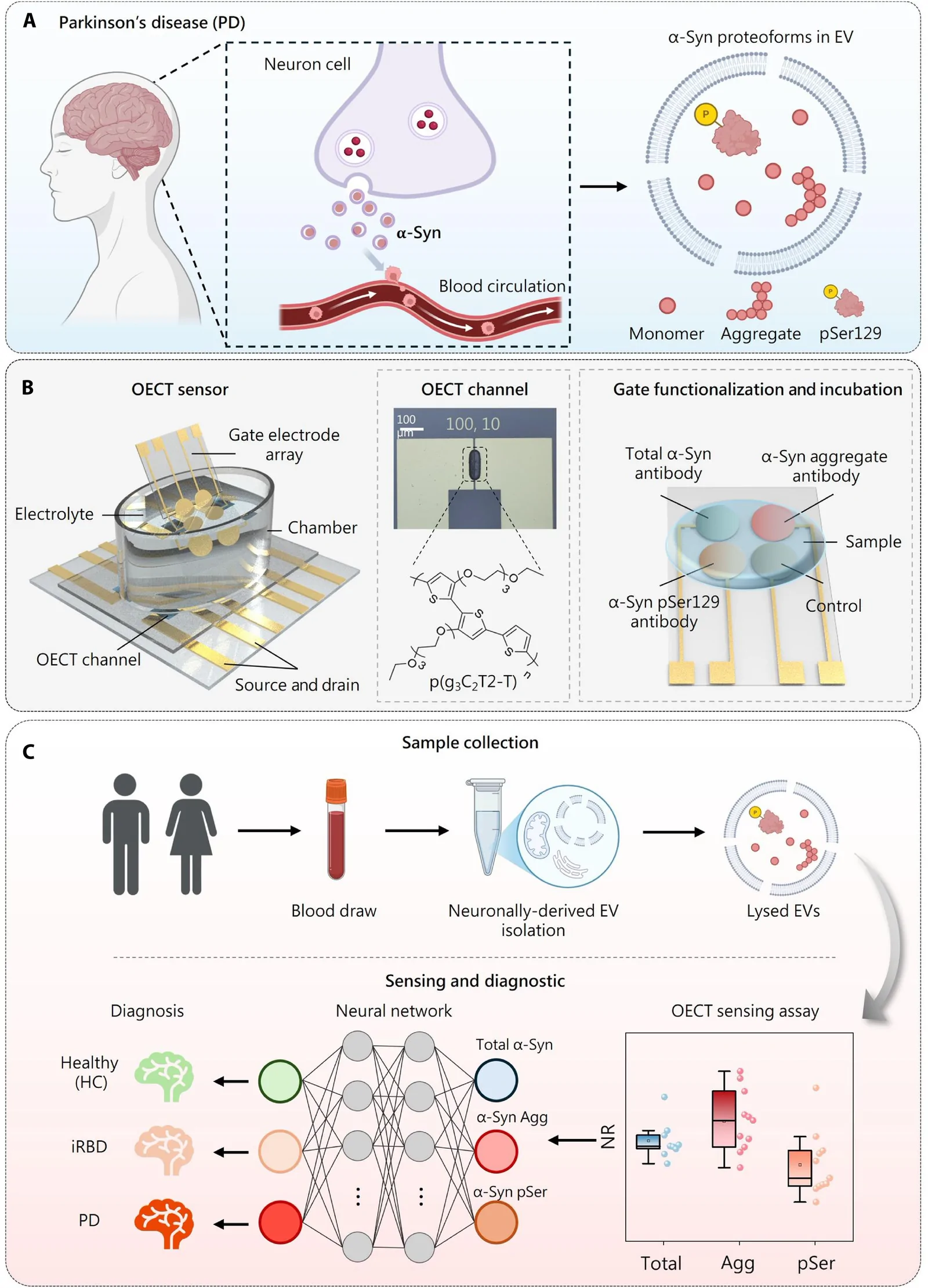
Multiplexed electronic detection of α-Syn proteoforms in neuronal EVs for PD diagnosis. (**A**) Misfolded α-Syn originating from degenerating neurons is released into circulation within neuronal EVs. These EVs carry total, aggregated, and pSer129 α-Syn proteoforms that reflect underlying brain pathology. (**B**) OECT sensor configuration used for multiplex detection. The device consists of patterned p-type organic mixed ionic-electronic conductor p(g_3_C_2_T2-T) (width, 100 μm; length, 10 μm), and functionalized gate electrodes bearing capture antibodies for total α-Syn, aggregated α-Syn, and pSer129 α-Syn. (**C**) Clinical workflow: serum is collected from participants, neuronal EVs are isolated via L1CAM enrichment and lysed, and α-Syn proteoforms are quantified by the OECT assay. Combined proteoform signatures are analyzed using a neural network classifier to differentiate healthy controls (HC), individuals with isolated iRBD (i.e., prodromal PD), and clinically diagnosed PD. Created in BioRender. Chang, T. (2026) https://BioRender.com/16sedwg.

The gate electrode array contained four individually addressable Au electrodes (diameter: 2.5 mm) functionalized with antibodies against total α-Syn (ab138501), aggregated α-Syn (ab209538), and pSer129 α-Syn (ab59264), respectively. The fourth electrode was modified with an anti–green fluorescent protein (GFP) antibody to serve as a noninteracting, negative control electrode. For clarity, we refer to the targets as total α-Syn, α-Syn Agg, and α-Syn pSer, and to their corresponding antibodies as total α-Syn Ab, α-Syn Agg Ab, and α-Syn pSer Ab.

During sensing, the functionalized array was incubated with serum-derived L1CAM^+^ EV samples (or buffer-spiked α-Syn standard solutions). Changes in OECT current before and after target exposure were recorded across all electrodes, yielding characteristic signatures for each sample. The resulting signal combinations were analyzed using a neural network classifier, which integrated responses from all three sensors to differentiate PD, iRBD, and HC samples (Fig. 1C).

### Turning gate electrodes into protein-capturing surfaces

To enable selective capture of α-Syn proteoforms, we engineered the gold gate electrodes into stable biofunctional interfaces using a polyethylene glycol (PEG)–based self-assembled monolayer (SAM) followed by covalent antibody immobilization (Fig. 2A). Cleaned Au electrodes (step 1) were immersed in ethanol containing a mixture of thiolated PEG chains [4 or 12 units of ethylene oxide; m-dPEG_4_-thiol (PEG4): m-dPEG_12_-thiol (PEG12) = 9:1] to form a mixed-length PEG SAM (step 2). PEG-based SAMs have been reported to exhibit antifouling properties (*26*, *27*), and the particular composition was selected to provide spatial accessibility for subsequent bioconjugation (*28*). The SAM was then activated in the solution of EDC [1-ethyl-3-(3-dimethylaminopropyl)carbodiimide] and NHS (*N*-hydroxysuccinimide) to generate reactive NHS esters (step 3), enabling covalent attachment of antibodies targeting α-Syn, α-Syn Agg, and α-Syn pSer and the GFP Ab. Upon drop-casting, amine groups on each antibody reacted with the activated surface to form amide bonds (step 4). Last, ethanolamine (ETA) was applied to quench unreacted esters and block nonspecific adsorption sites, producing a uniform and chemically stable protein-capturing surface (step 5).

**Fig. 2.**
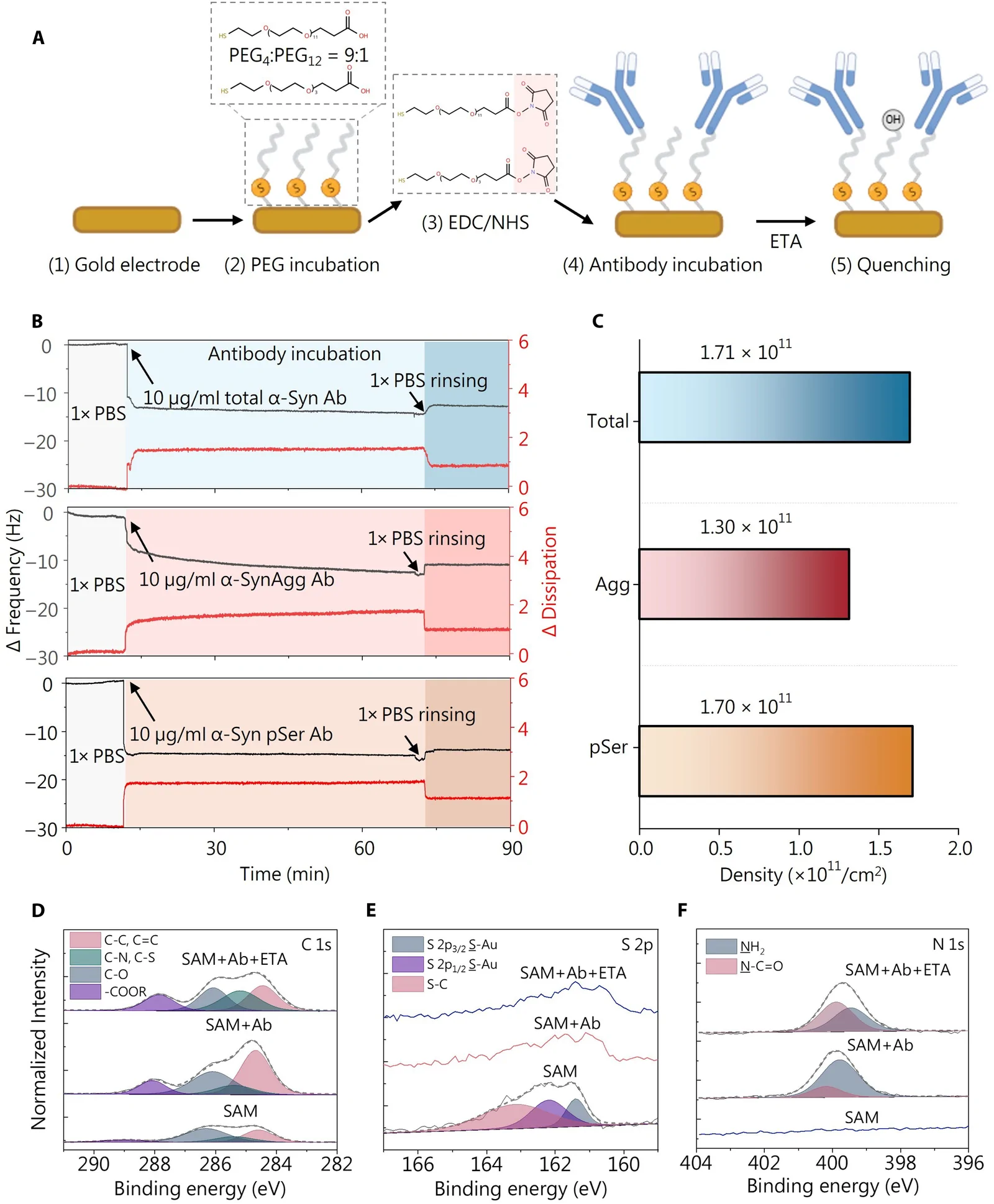
Physicochemical characterization of antibody-functionalized Au electrodes. (**A**) Illustration of the functionalization workflow, beginning with SAM formation on the Au gate electrode, followed by covalent immobilization of antibodies recognizing total α-Syn, aggregated α-Syn, or pSer129 α-Syn, and final blocking with ETA to passivate remaining reactive sites. (**B**) QCM-D dissipation and frequency traces demonstrating distinct mass increases associated with antibody binding, with subsequent PBS rinsing indicating stable attachment and removal of nonspecific adsorbates. (**C**) Antibody surface density calculated from QCM-D mass shifts, assuming a molecular weight of 150 kDa per antibody. (**D** to **F**) High-resolution XPS spectra (C 1s, S 2p, and N 1s) measured after SAM deposition, antibody coupling, and EA blocking. Increases in nitrogen- and sulfur-containing components, along with shifts in carbon signatures, confirm stepwise chemical modification of the Au surface and retention of antibody-associated elemental signatures. Created in BioRender. Chang, T. (2026) https://BioRender.com/hwxv7vf.

We validated the functionalization strategy using quartz crystal microbalance with dissipation monitoring (QCM-D) as well as x-ray photoelectron spectroscopy (XPS). PEG SAM bearing and EDC/NHS activated electrodes were placed into the QCM-D chambers containing phosphate-buffered saline (PBS; Fig. 2B). After baseline stabilization, antibody solution (10 μg/ml) was introduced for 5 min at 100 μl/min, followed by 1 hour for incubation and PBS rinse. The observed decreases in frequency signals and the increase in dissipation signals confirmed successful mass loading and viscoelasticity changes associated with antibody binding. On the basis of the QCM-D frequency shifts, we estimated mean surface density of 1.71 × 10^11^/cm^2^, 1.30 × 10^11^/cm^2^, and 1.70 × 10^11^/cm^2^ for total α-Syn Ab, α-Syn Agg Ab, and α-Syn pSer Ab, respectively (Fig. 2C).

XPS further verified the stepwise chemical assembly (Fig. 2, D to F). The high-resolution C1s spectrum of the PEG SAM-modified Au displayed characteristic peaks corresponding to C─C (∼284.8 eV), C─O (∼286.2 eV), and minor C─S (∼285.3 eV) bonds. Following antibody immobilization, the proportion of C─C increased and a new component at ∼288.5 eV emerged, consistent with peptide-bonded carbonyls from the antibody layer. Subsequent ETA blocking increased the C─O signal, reflecting its terminal hydroxyl moieties. In the S 2p region, a clear S─Au signature indicative of thiol-gold bonding was present after SAM formation, and its attenuation after antibody and ETA addition indicates that biological layers increasingly shield the underlying SAM from x-ray probing. The N 1s spectrum showed negligible signal on PEG SAM–only surfaces but revealed a pronounced peak near ∼400 eV after antibody and ETA deposition, confirming successful incorporation of nitrogen-containing biomolecules. Together, the QCM-D and XPS analyses confirm the formation of a well-defined PEG SAM, efficient antibody immobilization, and subsequent passivation, yielding robust protein-capturing gate electrodes as depicted in Fig. 2A.

### Electrode sensitivity to target proteins

To evaluate the sensing behavior of each functionalized gate electrode before clinical testing, we characterized their electrochemical and transistor-level responses to commercial α-Syn protein standards (Fig. 3). Electrochemical impedance spectroscopy (EIS) revealed distinct target-dependent signatures across the three α-Syn sensors. Sensors were preincubated in 1% Triton X-100, rinsed, and measured twice in PB to establish stable blank baselines, followed by incubation with 10 pM target proteins (GFP control with 10 pM total α-Syn) in Triton X-100. For the total α-Syn, α-Syn Agg, and α-Syn pSer sensors, Nyquist plots showed a change in their slope consistent with protein exposure, whereas the GFP control data remained unchanged (Fig. 3, A to D). Fitting EIS data to a circuit with a constant-phase element (*29*) (fig. S2, CPE in series with a resistor, R, inset of fig. S2), we find a reduction in gate capacitance (*C*_g_) in all three α-Syn sensors after target binding, which remained stable in the GFP control (fig. S3A), confirming selective bioelectrochemical modulation of the gate interface. Binding of α-Syn proteoforms also produced measurable shifts in the surface potential of the electrodes (φ_g_) measured using open-circuit potential (OCP) measurements (Fig. 3, A to D, bottom). For each α-Syn–targeting electrode, φ_g_ decreased after protein exposure, reflecting alterations in electrode interfacial potential caused by antibody-antigen interactions (Fig. 3, A to C, bottom).

**Fig. 3.**
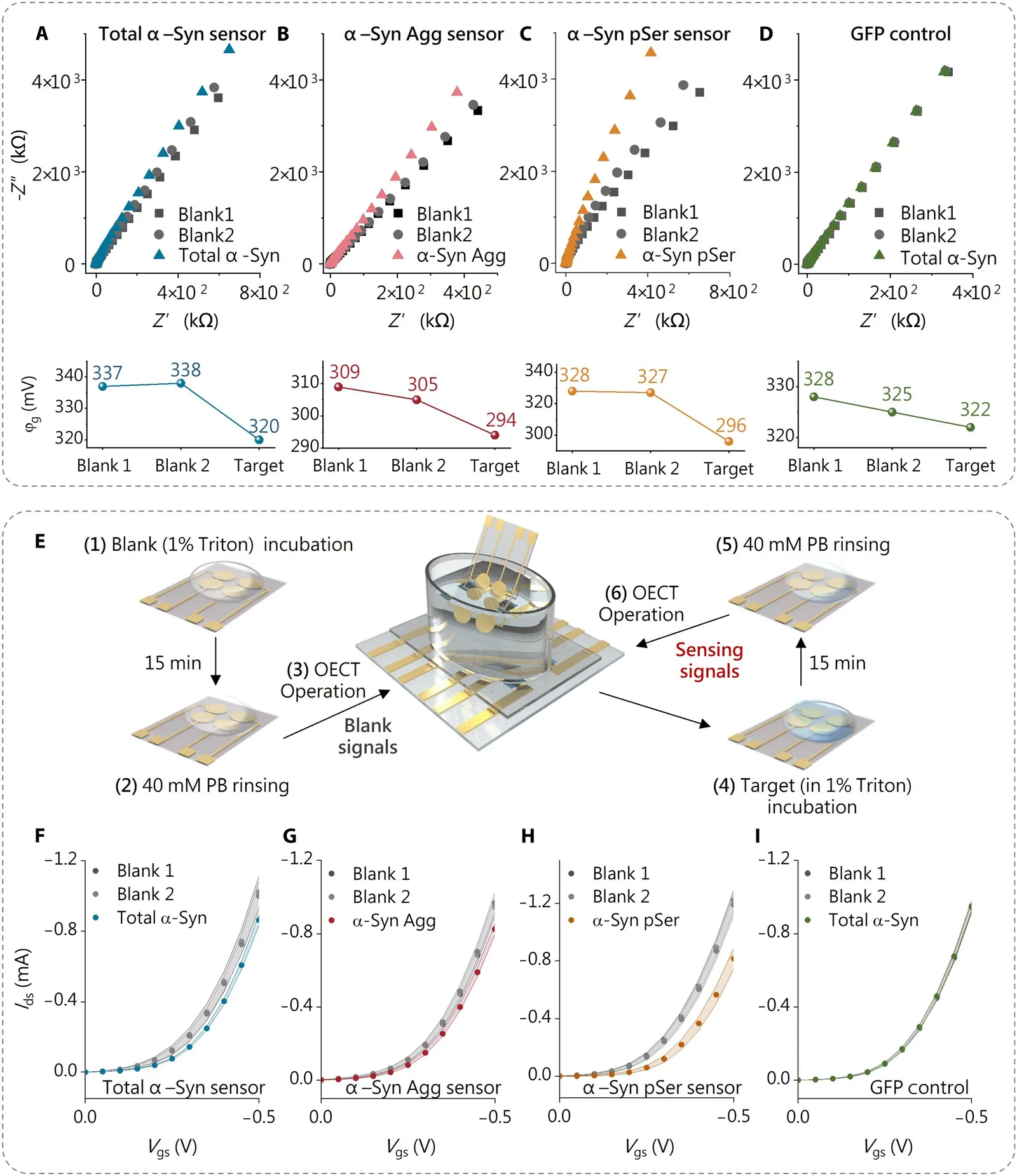
Electrochemical impedance- and transistor-based characterization of multiplexed sensors exposed to commercial α-Syn targets. (**A** to **D**) Nyquist plots (top) and the surface potential changes (bottom) of the (A) total α-Syn, (B) α-Syn Agg, (C) α-Syn pSer, and (D) GFP control sensors before (blank 1 and blank 2) and after exposure to their respective commercial α-Syn target proteins (GFP control to 10 pM total α-Syn) in 1% Triton X-100. (**E**) Workflow of the OECT sensing sequence, including blank incubation, rinsing, measurement, target exposure, final rinsing, and measurement. (**F** to **I**) Representative OECT transfer curves for the detection of 10 pM of (F) total α-Syn, (G) α-Syn Agg, and (H) α-Syn pSer with (I) GFP control response to 10 pM total α-Syn. *V*_ds_ = −0.5 V. Blank 1 and blank 2 curves almost overlap, indicating minimal drift during the measurements. *n* = 3.

We next analyzed how these changes in φ_g_ and *C*_g_ translate into transistor-level responses. In the OECT, the variations of gate electrochemical properties (i.e., Δφ_g_ and Δ*C*_g_) upon antigen binding change the portion of the *V*_gs_ that is transmitted to the channel (*30*–*32*). *V*_gs_ is distributed between the electrical double layers formed at the gate and channel interfaces with the electrolyte, and this distribution depends on their relative capacitances (*23*, *30*, *33*, *34*). Target binding produces two coupled electrochemical changes at the gate. First, the gate surface potential decreases by Δφ_g_, which shifts the gate voltage felt by the channel to lower values ($V_{\text{gs}}^{\text{eff},1}$). Second, its capacitance decreases by Δ*C*_g_, which means weaker doping at the channel ($V_{\text{gs}}^{\text{eff},2}$) (fig. S3B). More details are provided in the note S1.

For the p-type mixed conductor we chose to use in the channel, the reduction in gating is expected to decrease the measured drain current (*I*_ds_). We assessed the effect of commercial α-Syn targets on transistor-level responses. The OECT workflow (Fig. 3E and movie S1) included blank incubations (1% Triton X-100), PB rinsing, OECT blank measurements, target incubation (1 fM α-Syn), PB rinsing, and final OECT measurements, similar to the protocol developed for the electrode measurements. Sensors showed excellent baseline stability across five blank incubation cycles, with nearly overlapping transfer curves (fig. S4). Upon incubation with 1 fM protein targets, all three α-Syn sensors exhibited downward shifts in their transfer curves (Fig. 3, F to I), fully consistent with the EIS/OCP-derived detection mechanism described above. The GFP control exhibited no noticeable current modulation (Fig. 3I), confirming specificity. We investigated concentration-dependent OECT responses. As shown in fig. S5 (A to C), increased protein concentration produced progressively larger reductions in *I*_ds_. Corresponding normalized responses (NRs; fig. S5, D to F) confirmed excellent channel stability (reference electrode gated channel response <5%) and negligible cross-reactivity of GFP control electrodes. All three α-Syn sensors exhibited approximately linear calibration curves, enabling quantification across several orders of magnitude. The limits of detection (LOD), calculated as 3σ per slope, were 457 aM (6.39 fg/ml) for total α-Syn, 12.7 aM (0.18 fg/ml) for α-Syn Agg, and 11.3 aM (0.16 fg/ml) for α-Syn pSer. We quantified variability in sensor response by calculating the intrachip coefficient of variation (CV) across multiple target concentrations. For the total α-Syn sensor, the CV values ranged from ∼13 to 25%; for the α-Syn Agg sensor, from ∼6 to 38%; and for the α-Syn pSer sensor, from ∼9 to 40%, depending on the concentration (fig. S5, G to I). Higher CV values were observed at lower concentrations (e.g., 1 and 100 fM), which is likely due to the reduced signal-to-noise ratio near the detection limit. At higher concentrations (≥100 pM), the CV values decreased (typically <15%), indicating improved reproducibility when the sensing signal is higher. The intrachip variability across all concentrations can be attributed to our manual device preparation process, which leads to differences in gate electrode properties and its target capturing ability.

To understand whether OECT is superior to the electrode format, we performed a direct comparison using the gate electrodes biofunctionalized to detect α-Syn Agg and recording their electrochemical impedance spectra under conditions identical to the OECT. For EIS measurements, the functionalized gate electrodes were sequentially incubated with 1% Triton X-100 to obtain two baseline measurements (blank 1 and blank 2), followed by incubation with 100 fM and 10 pM α-Syn Agg (in 1% Triton X-100). The spectra were recorded in 40 mM PB using a three-electrode configuration over a frequency range of 0.1 Hz to 1 MHz and fitted using an R-CPE equivalent circuit to extract the *C*_g_. As shown in fig. S6 (A to C), the variation between blank 1 and blank 2 was minimal, indicating low baseline noise. Upon target binding, no signal was observed at 100 fM, whereas a measurable change in *C*_g_ emerged at 10 pM (fig. S6, A to D). The SNR for the EIS-based sensors was calculated as the ratio of the NR (of *C*_g_ change) at 10 pM to the NR variation between blank measurements (fig. S6F). For comparison, the SNR of the OECT platform was calculated from the normalized current response at 10 pM, derived from the calibration curves presented in fig. S5D (reproduced as fig. S6E), relative to blank variation shown in fig. S4D. OECT platform exhibited a nearly fourfold higher SNR (fig. S6F). This comparison also revealed a substantial difference in LOD: While EIS fails to resolve a signal at 100 fM, OECT measurements clearly detect responses at this concentration and extend into the sub-femtomolar regime (fig. S5). Together, these results demonstrate that the effective LOD of OECT is orders of magnitude lower than that of the gate electrodes operated alone under identical sensing conditions.

We performed a storage stability study using four independent sets of α-Syn Agg sensing gates and GFP control gates (each set comprising three α-Syn Agg sensors and three GFP controls). Following biofunctionalization, the electrodes were stored at 4°C in 1× PBS, consistent with the standard storage protocol used in this work. Freshly prepared sensors were evaluated through two consecutive blank measurements in 1% Triton X-100, followed by detection of 100 fM α-Syn Agg in the same medium. As shown in fig. S7 (A and C), the as-prepared α-Syn Agg sensors exhibited stable blank signals and a clear negative NR upon target binding, with an average value of 42% and an interchip CV of 8.81%. GFP controls remained close to baseline, confirming low background drift and target specificity. The remaining electrodes were then stored at 4°C in 1× PBS and tested after 7, 10, and 14 days using the same protocol. After 7 days of storage, the α-Syn Agg sensors still showed a clear response to the target although the signal decreased by ∼6% compared with freshly prepared electrodes (fig. S7, B and D), and the variability increased (CV = 39.61%). After 10 days, the target-induced response became negligible (fig. S7E), and no distinguishable sensing signal was observed after 14 days (fig. S7F). These results indicate that the functionalized gate electrodes maintain practical sensing capability for up to 7 days when stored at 4°C in PBS, but degrade thereafter, with near-complete loss of activity after 10 days. This decline is likely driven by gradual loss of antibody activity and/or degradation of the biofunctional interface during prolonged wet storage.

### Clinical validation of OECT sensors using patient-derived EVs

Considering these results, which highlight the potential of the multiplexed OECT platform for detecting α-Syn proteoforms at sub-femtomolar concentrations, well suited for low-abundance EV samples, we next evaluated sensor performance using patient-derived L1EVs. Serum samples collected from individuals diagnosed with PD and iRBD, as well as those from HC, were processed blindly for L1EV extraction. L1EVs were released from the capture matrix using a glycine buffer (pH 2.9) and immediately neutralized with tris buffer. Transmission electron microscopy images confirmed the presence of vesicles with the characteristic cup-shaped morphology and double-membrane structure, with an average diameter of ∼100 nm (fig. S8). Nanoparticle tracking analysis verified that most captured L1EVs were ∼100 nm in size (fig. S9). L1EVs were immunocaptured and lysed as described previously (*15*), with successful L1EV immunocapture from serum shown in fig. S10.

Following the workflow established for commercial protein standards, we first characterized the sensors using a representative clinical sample (iRBD no. 1). Because only 10 μl of sample was available, the lysate was diluted 1:50 in 1% Triton X-100 to obtain sufficient volume for electrode incubation. Two consecutive blank measurements confirmed stable baseline behavior. Incubation with the iRBD no. 1 sample produced capacitance increases in the total α-Syn, α-Syn Agg, and α-Syn pSer sensors (Fig. 4, A to C, and see fig. S11 for the fits), while the GFP control remained unchanged (Fig. 4D). This trend is opposite to the commercial protein case (Fig. 3), suggesting that the electrochemical properties of patient-derived EV cargo differ from those of purified proteins (see note S2). Zeta potential measurements revealed that the clinical α-Syn–containing sample carried a much weaker net charge (−6 mV) than the commercial α-Syn standards (−20 to −15 mV), indicating a higher tendency to form aggregates in solution (fig. S12). Such aggregated species generate a thicker, hydrated, and ion-permeable biolayer on the electrode surface, which increases the apparent interfacial capacitance. To probe specificity, we spiked the iRBD no. 1 sample with 10 pM pSer129 α-Syn. The total α-Syn and α-Syn pSer sensors showed further capacitance increases (Fig. 4, A and C), whereas the α-Syn Agg sensor remained unchanged (Fig. 4B), confirming proteoform-selective responses within the complex clinical matrix.

**Fig. 4.**
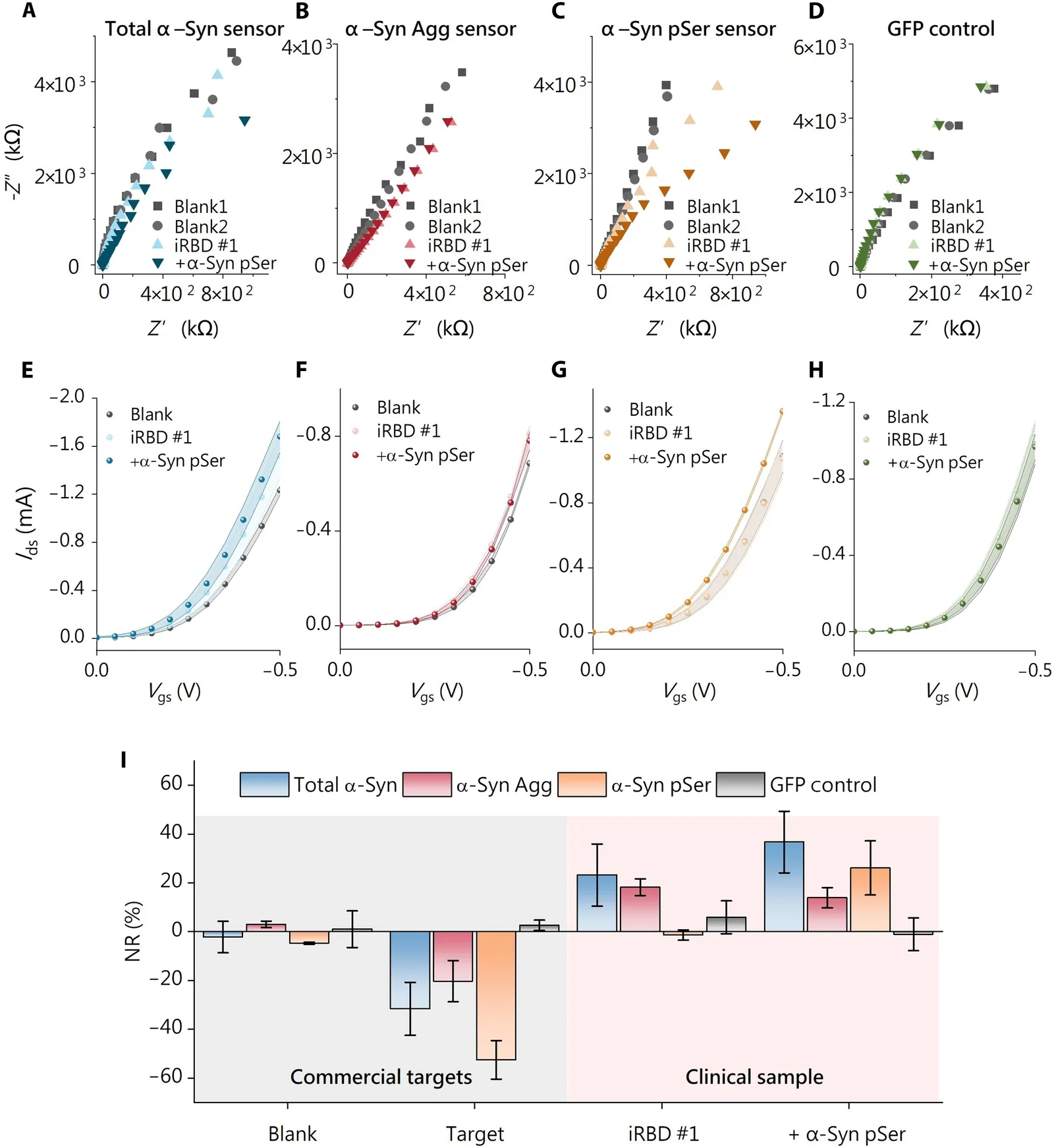
Electrochemical impedance and OECT characterization of α-Syn sensors exposed to clinical samples. (**A** to **D**) Nyquist plots of the (A) total α-Syn, (B) α-Syn Agg, (C) α-Syn pSer, and (D) GFP control sensors measured in 40 mM PB. (**E** to **H**) Transfer curves (*V*_ds_ = −0.5 V) of the (E) total α-Syn, (F) α-Syn Agg, (G) α-Syn pSer, and (H) GFP control sensors. (**I**) Normalized OECT responses (NR) comparing the behavior of each α-Syn sensor toward commercial α-Syn standards (left, gray background) and toward the clinical sample (right, pink background). Total α-Syn, α-Syn Agg, and α-Syn pSer channels show target-specific responses, while the GFP control remains stable.

While the capacitance of all gate electrodes increased with the target, the φ_g_ of the total α-Syn and α-Syn pSer sensitive electrodes decreased after exposure to the iRBD no. 1 sample and after pSer129 spiking (fig. S13, A and B). The effect of the combined electrochemical changes on the $V_{\text{gs}}^{\text{eff}}$ is illustrated in fig. S13C where φ_g_ and *C*_g_ exert opposing influences on the resulting channel current. A decrease in φ_g_ is expected to lower $V_{\text{gs}}^{\text{eff}}$ (i.e., $V_{\text{gs}}^{\text{eff},1}$) and therefore reduce *I*_ds_, whereas an increase in $C_{g}$ reduces the voltage drop at the gate electrode and increases $V_{\text{gs}}^{\text{eff}}$, raising *I*_ds_. We probed these changes in device terminal potentials in real time for an OECT before and after its exposure to the iRBD no. 1 sample using an integrated OCP-OECT setup as shown in fig. S14 and discussed in note S2.

For the clinical samples, the capacitance effect clearly dominated, yielding a net increase in *I*_ds_. After incubation with the iRBD no. 1 sample, transfer curves for the total α-Syn (Fig. 4E) and α-Syn Agg sensors (Fig. 4F) shifted upward, consistent with *C*_g_-driven increases in $V_{\text{gs}}^{\text{eff}}$. In contrast, the α-Syn pSer (Fig. 4G) and GFP control sensors (Fig. 4H) showed minimal response, indicating lower pSer129 abundance in this sample. Subsequent addition of pSer129 α-Syn produced clear increases in the current of the total α-Syn (Fig. 4E) and α-Syn pSer (Fig. 4G) sensors, confirming their specificity within the clinical matrix. NRs for both commercial protein standards and the iRBD no. 1 sample (*V*_ds_ = *V*_gs_ = −0.5 V) are summarized in Fig. 4I. Together, these measurements demonstrate that the multiplexed OECT platform provides stable, specific, and interpretable responses in patient-derived neuronal EV samples, validating its suitability for blinded clinical testing across the full study cohort.

EIS and OECT measurements with the first clinical sample (iRBD no. 1) established the sensing mechanism and suggested that the relative abundance of α-Syn proteoforms differs. Building on these observations, we next evaluated a set of nine known clinical samples, three PD, three iRBD, and three HC, each diluted 50-fold in 1% Triton X-100. Representative transfer curves and NRs for one sample from each diagnostic group are shown in Fig. 5; the remaining six samples are presented in figs. S15 and S16.

**Fig. 5.**
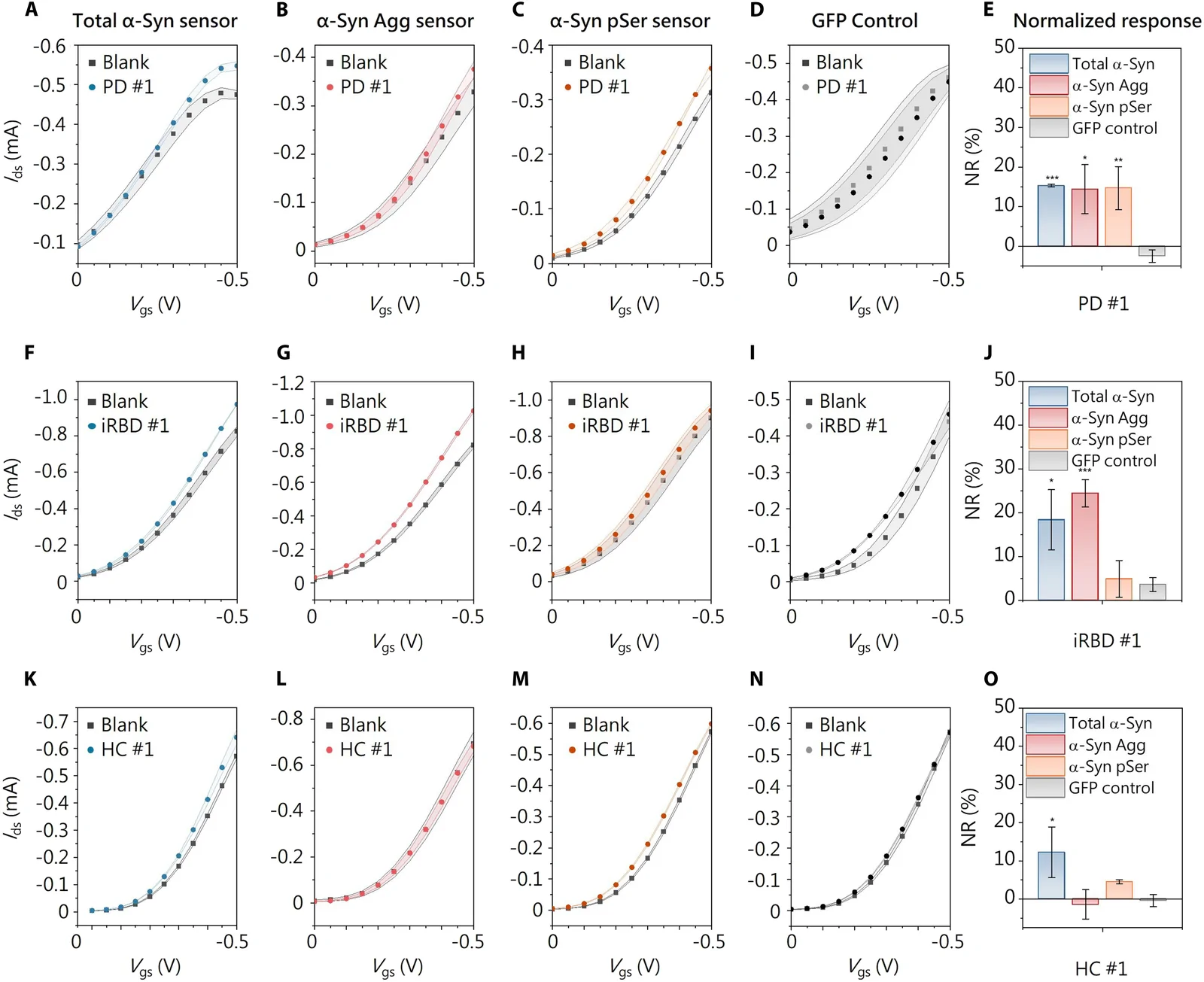
Multiplexed OECT sensor response to clinical samples. Representative (**A** to **E**) PD sample, (**F** to **J**) iRBD sample, and (**K** to **O**) HC sample. Transfer curves were recorded at *V*_ds_ = −0.5 V and NRs were calculated at *V*_ds_ = *V*_gs_ = −0.5 V. The error bars and shaded bands represent measurement from three independent gate electrode arrays (*n* = 3) exposed to the same sample. Blue, red, and orange bars correspond to total α-Syn, α-Syn Agg, and α-Syn pSer sensors, respectively, while the gray bar indicates the GFP control.

For all PD samples, the total α-Syn, α-Syn Agg, and pSer sensors demonstrated clear and consistent responses that significantly differed from the controls, which exhibited minimal signal changes. Sensor-to-sensor variation was low (*n* = 3 independent gate arrays), reflected in narrow error bands, confirming excellent electrode reproducibility. In contrast, iRBD samples exhibited strong responses only on the α-Syn Agg sensors, while the α-Syn pSer sensors did not differ appreciably from controls. Last, in HC samples, neither the α-Syn Agg nor α-Syn pSer sensors showed significant deviations from the control electrode.

Together, these initial observations suggest that both α-Syn Agg and α-Syn pSer levels are elevated in PD samples, whereas only α-Syn Agg is distinctly elevated in iRBD. Healthy controls show no detectable increases in any α-Syn subtype. These findings provide an early signature of proteoform-specific differences that distinguish prodromal and clinical stages of synucleinopathy and motivate blinded validation across the full patient cohort.

### Blind testing of clinical samples to assess OECT diagnostic accuracy

To assess the translational performance of the platform, we conducted a blinded evaluation of 57 clinical samples, with diagnostic identity concealed from the operator (Table 1). Classification criteria for PD, iRBD, and HC were defined as a priori based on our preliminary clinical testing. Samples were designated as PD when both α-Syn Agg and α-Syn pSer responses differed significantly from controls (*P* < 0.05). Samples were classified as iRBD when aggregate responses were significantly different from controls, even if α-Syn pSer responses were not. HC was defined by the absence of significant differences in either α-Syn Agg or α-Syn pSer responses relative to controls. As total α-Syn is broadly expressed in the body, responses from total α-Syn sensors were treated as a positive control for successful EV capture and lysis.

**Table 1.** A summary of clinical sample characteristics and the clinical sample tests. MoCA, Montreal Cognitive Assessment, a screening test for cognitive impairment; IQR, interquartile range.

	HC	iRBD	PD
No. of individuals	22	25	19
Used for blind tests	19	22	16
Female	6	1	1
Male	16	24	18
Age, mean (SD)	64.6 (12.3)	69.5 (6.0)	64.8 (10.3)
MoCA, mean (SD)	25.5 (2.8)	27.9 (1.3)	25.7 (2.6)
Total α-Syn, median (IQR), pg/ml*	12.65 (3.4)	42.05 (14.0)	32.82 (12.4)
NR total α-Syn, mean, %†	8.7	18.2	14.6
NR aggregated α-Syn, mean, %†	7.3	18.7	21.1
NR α-Syn pSer 129, mean, %†	4.8	5.3	19.4
Correctly identified in blind tests by OECT sensors, *n*	17	18	15

* Measurements of total α-Syn in L1EV using MSD electrochemiluminescence.

† Quantification of α-Syn using the OECT assay.

Sensor readouts for all blinded samples were revealed post-unblinding (figs. S17 to S21) and interpreted using the predefined classification criteria, with an NR of 12% (derived from the mean NR of GFP controls) set as the cutoff for significance. The heatmap in Fig. 6A demonstrates distinct biomarker response patterns across the two disease-associated clinical groups. The data shown in Fig. 6A also include nine samples characterized in Fig. 5 and figs. S15 and S16. In total blind tests, 15 of 16 PD samples, 18 of 22 iRBD samples, and 17 of 19 HC samples met our classification criteria, yielding an overall multiplexed sensor accuracy of 87.7%, with a sensitivity of 86.8% and a specificity of 89.5% (Table 1).

**Fig. 6.**
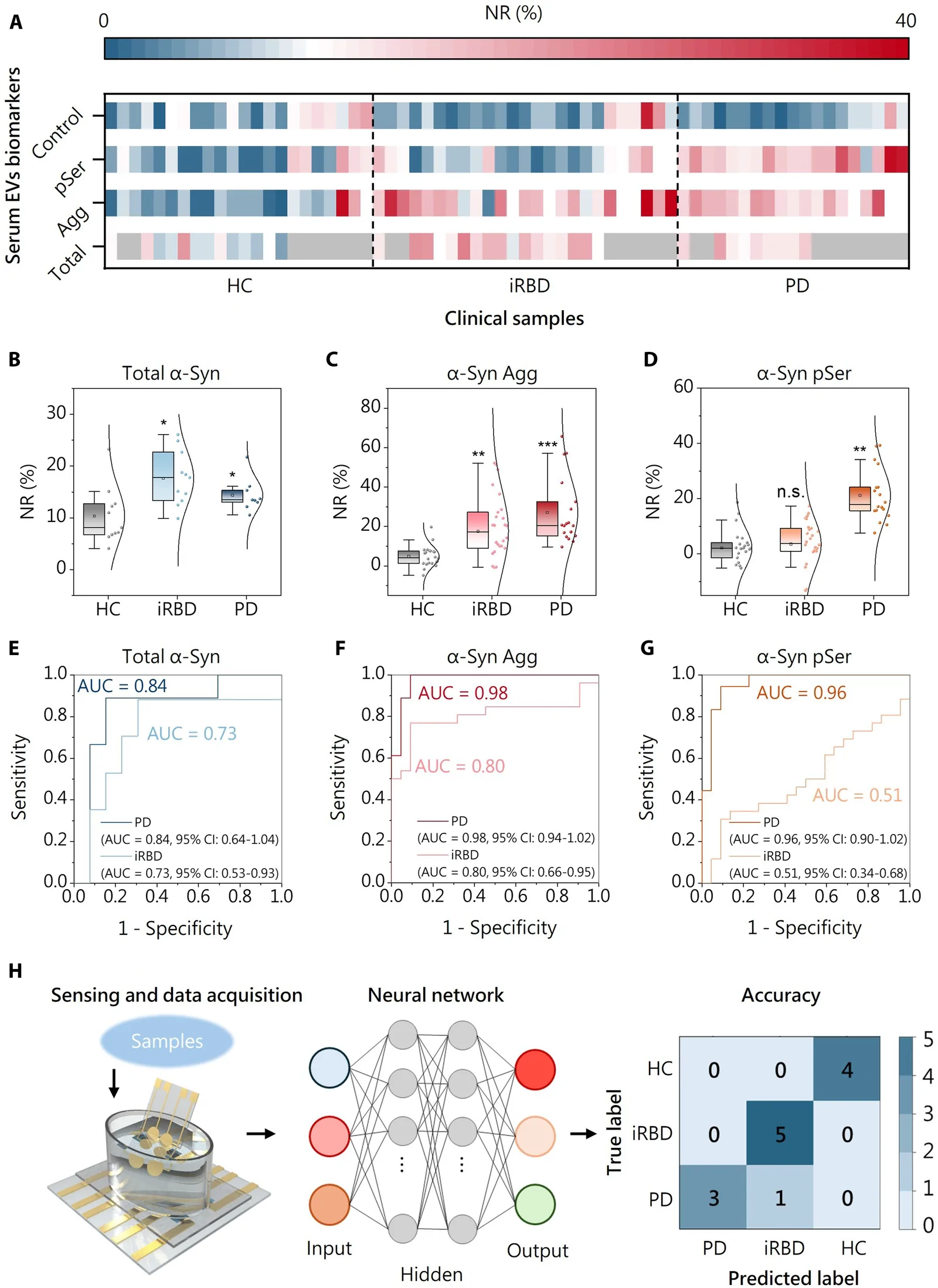
Blinded diagnostic performance of the multiplexed OECT across the full clinical cohort. (**A**) Heatmaps showing the levels of total α-Syn, α-Syn Agg, and α-Syn pSer in serum EVs from all clinical samples (*n* = 66), grouped by diagnostic category (HC, iRBD, and PD). Gray areas stand for no tested data. (**B** to **D**) Distribution of NR values for (B) total α-Syn, (C) α-Syn Agg, and (D) α-Syn pSer sensors across the three diagnostic groups. NR values were calculated at *V*_ds_ = *V*_gs_ = −0.5 V. (**E** to **G**) Receiver operator characteristic (ROC) curves for discriminating PD from HC using (E) total α-Syn, (F) α-Syn Agg, and (G) α-Syn pSer. (**H**) Schematic of the neural network–based classifier and confusion matrix summarizing diagnostic accuracy across all blinded samples.

As an orthogonal benchmark, total α-Syn concentrations were measured using a Meso Scale Discovery (MSD) electrochemiluminescence assay. OECT responses showed similar distributions to MSD results (fig. S22, A and B) and correlation analysis (*R*^2^ = 0.53; fig. S22C) confirmed quantitative concordance between the two methods. Higher OECT NR values consistently aligned with elevated α-Syn concentrations quantified by MSD.

Box plot analyses in Fig. 6 (B to D) highlight the significant marker concentration differences across disease stages. Total α-Syn and α-Syn Agg responses were significantly elevated in both PD and iRBD relative to HC (Fig. 6, B and C). In contrast, α-Syn pSer showed a PD-specific increase, with levels significantly higher in PD than in iRBD or HC (Fig. 6D), highlighting its role as a PD-specific marker. ROC curve analysis further quantified diagnostic performance (Fig. 6, E to G). Total α-Syn and α-Syn Agg differentiated PD and iRBD from HC (AUC = 0.98, 95% CI: 0.94 to 1.02 for PD; AUC = 0.80, 95% CI: 0.66 to 0.95 for iRBD), while α-Syn pSer effectively distinguished PD from iRBD with high accuracy (AUC = 0.96, 95% CI: 0.90 to 1.02). These findings validate the reliability of both the OECT sensors to detect individual markers and demonstrate that each α-Syn proteoform provides complementary diagnostic information, with α-Syn Agg acting as a broader synucleinopathy marker and α-Syn pSer serving as a PD-specific indicator.

To integrate multiplexed sensor outputs into a single diagnostic decision, we implemented a supervised classifier based on a multilayer perceptron (MLP) neural network (Fig. 6H). Input features included normalized sensor readouts for total α-Syn, α-Syn Agg, and pSer, standardized using *z*-score normalization. The MLP architecture consisted of two hidden layers [16 and 8 neurons, rectified linear unit (ReLU) activation], and a three-neuron SoftMax output layer. Trained using Adam optimization over 2000 iterations, the classifier achieved 90% accuracy on a stratified 20% test set. Precision, recall, and F1 score metrics indicated strong performance for HC and iRBD, with slightly reduced recall for PD. Principal components analysis PCA) confirmed group separation in reduced-dimensional space, demonstrating distinguishable clustering trends among the three groups.

## DISCUSSION

The detection of L1EV protein content that reflects brain pathology offers a means of developing a liquid biopsy for PD and related disorders. This requires an appropriate sensing platform for the detection of rare but disease-relevant proteoforms. We developed an OECT sensor that successfully detected α-Syn pSer and α-Syn Agg forms in L1EVs isolated from serum. The OECT sensing platform was designed using a high-performance semiconducting polymer and a multiplexed gate electrode array. The multiplexed gate electrodes achieved the detection of 3 α-Syn subtypes simultaneously. We developed a standard for sample classification based on both previous clinical research and the pilot results acquired from our experiments, demonstrating an accuracy of 87.7%, with a sensitivity of 86.8% and a specificity of 89.5% in detecting synucleinopathy in clinical samples. The ability to detect both pSer129 α-Syn and aggregated α-Syn enabled us to capture distinct changes in these proteoforms across the prodromal and clinical stages of PD. The composite marker constructed from several α-Syn proteoforms significantly improves the diagnostic accuracy compared to the performance of a single marker. Thus, our device can serve as a platform to further explore the most relevant combination of biomarkers in PD diagnostics.

Despite the growing interest in phosphorylated α-Syn as a disease-relevant biomarker, there is currently no established clinical evidence demonstrating that pSer129 α-Syn levels in L1EVs can reliably distinguish PD from iRBD. Previous studies using conventional assays, including our prior MSD-based study (*15*), were limited by insufficient sensitivity, with most clinical samples falling below the detection range. This limited sensitivity may have obscured potentially informative differences in EV-associated pSer129 α-Syn across disease stages. In contrast, the ultrahigh sensitivity of the OECT platform enables the quantification of trace-level L1EV-associated pSer129 α-Syn and supports the utility of this biomarker in differentiating iRBD from PD. As a flexible platform, the sensor can be readily extended to other biomarkers by customizing the biorecognition units on the gate electrodes.

Despite the promise of this study, it is important to note that the clinical sample size is small, and further validation of the assay performance and sensitivity is required in a larger independent cohort. From the system side, the best electrolyte condition for sensing may not be the best for the OECT channel material. For example, the electrolyte with a lower ionic strength and a longer Debye length helps more with the sensing signal translation, but that may cause an improper ionic environment for doping the channel material. The floating gate OECT setup, also known as the extended gate OECT, has been proven to be an effective way to solve the problem. By adding a floating gate, the sensing gate electrode (e.g., antibody functionalized gate electrode) and the gate electrode (e.g., Ag/AgCl electrode) for OECT operation can be set into two separate chambers, and in each chamber, the most suitable electrolyte can be used. This ensures both the efficiency of OECT operation (doping and de-doping) and the sensitivity of the sensors. So far, the variability inherent to hand-fabricated sensors remains a challenge, manifesting as sensor-to-sensor variation in output. This issue could be mitigated by introducing automated fabrication processes that precisely control parameters such as the volume of the antibody solution and the positioning of the electrode during biofunctionalization. Such improvements would help reduce variability and enhance reproducibility across devices. We also note that it should be possible to quantify the target proteins with sensors relying on capacitance changes. Future work could incorporate matrix-matched calibration strategies (e.g., spiking α-Syn into EV lysates or serum-derived matrices), a buffer optimization that can allow for a concentration-dependent OECT response or refined models that account for interfacial heterogeneity and aggregation effects. For truly portable sensors, suitable for future use at point of care, the EV capture and release will need to be integrated using microfluidics (*35*), achieving a multiparameter sensing platform.

## MATERIALS AND METHODS

### Materials

Sodium phosphate monobasic monohydrate (NaH_2_PO_4_·H_2_O) and sodium phosphate dibasic heptahydrate (Na_2_HPO_4_·7H_2_O) were purchased from Sigma-Aldrich and used to prepare the 40 mM PB solution (pH 7.4) by mixing 9.837 mM NaH_2_PO_4_·H_2_O with 30.163 mM Na_2_HPO_4_·7H_2_O in deionized water (DI water). Thiol-dPEG12-acid (PEG_12_), thiol-dPEG4-acid (PEG_4_), Triton X-100, ETA, EDC, NHS, hydrogen peroxide (H_2_O_2_), potassium hydroxide (KOH), and PBS (pH 7.4) were purchased from Sigma-Aldrich and used as received. Anti-GFP antibody, anti–α-Syn total antibody (ab138501), anti–α-Syn aggregates antibody (ab209538), anti–α-Syn pSer129 antibody (ab59264), α-Syn total protein, α-Syn protein aggregates (type 2, mixture of oligomers and fibrils), and α-Syn pSer129 protein were purchased from Abcam. p(g_3_C_2_T2-T) was synthesized according to a procedure reported previously (*23*).

### Device fabrication

OECTs and gate electrode arrays were fabricated on glass substrates using standard photolithography and parylene C peel-off techniques. Briefly, once the glass wafers were cleaned with piranha solution, they were spin coated with LOR 5B and S1813 photoresist. The coated wafers were then exposed to ultraviolet light using a contact aligner to create Au electrode patterns and interconnection pads. The photoresist patterns were then generated with the MF-319 developer, followed by metal sputtering of 10 nm of Cr and 100 nm of Au. The wafers were processed by a standard lift-off procedure using *N*-methylpyrrolidone. Next, the OECT wafers were deposited with two parylene C layers, and the gate electrode wafers were deposited with only one parylene C layer, followed by coating the layer of the AZ-10XT photoresist. After exposure, the wafers were developed using the AZ726 developer. Last, the wafers were etched so that the channel areas were opened. p(g_3_C_2_T_2_-T) from a chloroform solution (5 mg/ml) was spin coated (800 rpm; 45 s) on OECT channels. All devices were rinsed with DI water before use.

### Biofunctionalization of gate electrodes

The Au electrodes were first cleaned using a 50 mM KOH and 25% H_2_O_2_ solution for 15 min, then rinsed with DI water. The mixed PEG solution was prepared in absolute ethanol containing 1 mM PEG_4_ and 9 mM PEG_12_. The electrodes were immersed in the mixture overnight, then rinsed and sonicated with absolute ethanol for 5 min and dried under an N_2_ stream. Then, the electrodes were immersed in the EDC (5 mg/ml) and NHS (1 mg/ml) solution (in 1× PBS) mixture for 30 min for activation and stabilization. The electrodes were then incubated for 1 hour with antibodies (anti–α-Syn total, anti–α-Syn aggregate, anti–α-Syn pSer129, and anti-GFP) at 10 mg/ml in 1× PBS, then rinsed with 1× PBS. Each antibody solution was drop-cast on the corresponding electrode, and the solution was 40 μl, enough to cover one electrode only. Last, the electrodes were quenched by immersing in 1 M ETA for 20 min and rinsed with 1× PBS. Antibody-functionalized gate electrodes were stored at 4°C in 1× PBS until use.

### Quartz crystal microbalance with dissipation monitoring

The QCM-D measurements were performed using a QSense analyzer (QE401; Biolin Scientific). The piezoelectrically active gold sensor (0.7854 cm^2^) was first functionalized with PEG SAM under the same conditions explained above and then loaded into the QCM-D chamber. The QCM-D was calibrated, and the signals, including the frequency change (Δ*f_n_*) and dissipation change (Δ*D*), were stabilized in 1× PBS solution. Then, the EDC (5 mg/ml)/NHS (1 mg/ml) solution (in 1× PBS) was pumped into the chamber with a flow rate of 100 μl/min for 5 min. The pump was stopped for 30 min, and then 1× PBS was pumped into the chamber for 5 min to rinse the sensor surface. Next, α-Syn total antibody (10 mg/ml in 1× PBS) was pumped into the chamber and then incubated for 1 hour, followed by 1× PBS rinsing. Last, 1 M ETA (in 1× PBS) was pumped into the chamber and then incubated for 20 min, followed by 1× PBS rinsing. All QCM-D data were recorded at the seventh overtone (*n*). The Sauerbrey equation was applied to quantify the mass accumulating on the sensor (Δ*m*) during the functionalization steps

$$\Delta m=-17.7\times \frac{\Delta f_{n}}{n}$$

where *n* is the overtone number selected for the calculations and −17.7 is a constant calculated based on the resonant frequency, active area, density, and shear modulus of the quartz crystal sensor. The mass of the binding molecules on the sensor surface was then estimated using their molecular weight.

### X-ray photoelectron spectroscopy

XPS was performed using a Kratos AXIS Supra instrument equipped with a monochromatic Al Kα x-ray source (1468.6 eV). We operated the source at 75 W under ultrahigh-vacuum conditions (∼10^−9^ mbar). The spectra were recorded in a hybrid mode using electrostatic and magnetic lenses and an aperture slot of 300 μm × 700 μm. The survey and high-resolution spectra were acquired at fixed analyzer pass energies of 80 and 20 eV, respectively. We mounted the samples in a floating mode to avoid differential charging. The spectrum was then acquired under charge-neutralization conditions. We calibrated the spectra to a reference of C 1s at 284.8 eV. The Tougaard method was used for background subtraction, which was deconvoluted using Gaussian and Lorentzian methods with XPSPEAK41.

### Electrochemical measurements

The electrochemical characteristics of α-Syn sensors were investigated before and after the target protein incubation by EIS. α-Syn sensors were used as working electrodes, and EIS was performed using a conventional three-electrode setup and a potentiostat (PalmSens4). A platinum wire and a leakless Ag/AgCl electrode were used as the counter and reference electrodes, respectively. Measurements were carried out in 10 ml of 40 mM PB solution (pH 7.4). EIS signals were recorded at a 0 V DC voltage versus sensors’ OCP and an AC modulation of 10 mV over a frequency range of 0.1 to 1 MHz. Before starting the EIS measurement, a 60-s OCP was first measured. First, the functionalized sensor was incubated with 1% Triton X-100 (in 1× PBS) for 15 min, followed by rinsing three times with 40 mM PB. Then, the sensor was immersed in the 40 mM PB solution for the EIS characterization, and the OCP and the Nyquist plot were recorded as the first blank signals. Second, the same sensor was taken out and incubated again with 1% Triton X-100 for 15 min, followed by rinsing three times with 40 mM PB. Then, the sensor was immersed in the 40 mM PB solution for the EIS characterization, and the OCP and the Nyquist plot were recorded as the second blank signals. After confirming there was no drift between the first and the second blank signals, the same sensor was then incubated with α-Syn target (total/Agg/pSer/ clinical samples) solution (in 1% Triton X-100) for 15 min, followed by rinsing three times with 40 mM PB. Then, the sensor was immersed in the 40 mM PB solution for the EIS characterization, and the OCP and the Nyquist plot were recorded as the sensing signals.

### Clinical sample collection

Blood samples were obtained from participants in the Oxford Discovery cohort during clinical assessments, which were approved by the South Central - Oxford A Research Ethics Committee (IRAS188167). Written consent was obtained from all participants, and all protocols followed the principles of the Declaration of Helsinki. Serum was isolated, aliquoted, and stored at −80°C until further use. All samples were processed in a blinded manner. Serum samples and clinical data were collected from individuals with iRBD (*n* = 25) or PD (*n* = 19), and HC (*n* = 22), as summarized in Table 1. Participants were recruited from July 2013 through February 2024. Inclusion criteria for iRBD were confirmation by video-assisted polysomnography. PD patients were included based on clinical diagnostic criteria. HC without significant comorbidities or relevant family history were included. Full details of the discovery cohort and full inclusion/exclusion criteria have been described previously (*36*).

### L1EV isolation

For EV isolation, a three-step sequential spin (300*g* for 10 min, 2000*g* for 20 min, and 10,000*g* for 30 min) was used to remove cellular debris, protein aggregates, and fatty material from the serum. An appropriate amount of supernatant (250 μl for commercial electrochemiluminescence plate and 40 μl for OECT sensor), i.e., precleared serum, was transferred to protein low-binding tubes (Eppendorf) for immunocapture using anti-L1CAM immunobeads. Anti-L1CAM antibodies were preconjugated to magnetic beads coated with a zwitterionic poly(carboxybetaine methacrylamide) (pCBMA) polymer brush, as previously described and validated (*15*, *37*). This highly hydrated antifouling coating was used to reduce nonspecific adsorption in complex biofluids during L1EV immunocapture. The immunobeads were incubated at 4°C overnight on a rotating mixer, and bead-exosome complexes were collected by magnetic separation and washed successively with 0.05% Tween-20 in PBS (PBST) and PBS. Isolated L1EVs were lysed in 1% Triton X-100 in PBS with protease inhibitors for 30 min at room temperature for protein quantification.

### Western blot

EVs were isolated by anti-L1CAM immunobeads (with anti-CD9 immunobeads serving as a positive control for generic exosome capture and anti-HA immunobeads as a negative control) lysed in LDS buffer (Thermo Fisher Scientific). Subsequently, the lysates underwent separation via SDS–polyacrylamide gel electrophoresis, followed by transfer onto polyvinylidene fluoride membranes (Invitrogen). Immunoblotting was then performed using antibodies against syntenin-1 (ab133267, Abcam), CD9 (CBL162, Millipore), CD81 (sc-5275, Santa Cruz), NCAM (MA5-11563, Invitrogen), and L1CAM (ab80832, Abcam), all of which were diluted at a ratio of 1:1000. After incubation with a horseradish peroxidase–conjugated secondary antibody (GE Healthcare) diluted 1:10,000, immunodetection was carried out via chemiluminescence using a ChemiDoc system (Bio-Rad).

### Transmission electron microscopy

Transmission electron microscopy (TEM) was used to characterize the morphology and shape of exosomes eluted from pCBMA-functionalized magnetic beads. To release the captured EVs, 20 μl of glycine buffer (pH 2.9) was added, followed by immediate neutralization using 20 μl of tris buffer (pH 9.5). A 10-μl aliquot of the eluted sample was applied to a freshly glow-discharged carbon/formvar-coated 300-mesh copper grid for 2 min. Excess liquid was removed with filter paper, and the grid was negatively stained with 2% aqueous uranyl acetate for 10 s, then blotted and air-dried. Imaging was performed using a TEM operating at 120 kV, equipped with a Gatan OneView CMOS camera.

### MSD electrochemiluminescence

L1EVs immunocaptured from 250 μl of precleared serum were lysed in PBS containing 1% Triton X-100, supplemented with protease and phosphatase inhibitor cocktail. The lysates were collected after incubation for 30 min at room temperature with shaking. Total L1EV α-Syn was measured using MSD electrochemiluminescence U-PLEX assay (K1515NR-2, MSD).

### OECT measurement and statistical analysis

We used a Keithley Source Meter operated using a customized LabVIEW code to apply drain voltage (*V*_ds_) and gate voltage (*V*_gs_), and to get drain current (*I*_ds_) and gate current (*I*_gs_) under ambient conditions. A glass well was glued using polydimethylsiloxane (PDMS) on top of the channels and filled with 3 ml of 40 mM PB. The stability of the OECT was first tested by using a gold coil reference electrode, with *V*_ds_ applied between 0 and −0.5 V and *V*_gs_ between 0 and −0.5 V. After making sure the channel was stable, the sensing measurements started. For commercial sample tests, the sensor is first incubated with 1% Triton X-100 as the blank solution for 15 min, then rinsed with 40 mM PB, followed by immersing into the OECT electrolyte (40 mM PB) and obtaining the transfer curves (blank signals). After that, the sensor was taken out and incubated with 1 fM (14 pg/ml) target solution (in 1% Triton X-100) for 15 min and then rinsed with 40 mM PB and then immersed in the electrolyte to get the transfer curves (sensing signals; see movie S1).

Similar to commercial sample tests, for clinical sample tests, antibody-functionalized gate electrodes were first incubated for 15 min in 1% Triton X-100 followed by rinsing, then immersed in the electrolyte to get blank signals. The same electrodes were incubated for 15 min with a 30-μl 1% Triton X-100 solution containing target proteins. After thoroughly rinsing with 40 mM PB, the electrodes were used to get OECT sensing signals. Approved IBEC projects—21IBEC043, Polymer based Bioelectronic Diagnostics and 23IBEC026, Evaluation of Electronic Chips in Physiological Medium—were used to operate patient samples in this work.

The NR of the devices was calculated using the following equation

$$\text{NR}=\frac{I_{\mathrm{ds}}-I_{0}}{I_{0}}$$

where *I*_ds_ is the current response of the sensor after the gate was exposed to the protein/EV solution, and *I*_0_ is the current before exposure (i.e., blank). The *t* tests were conducted to compare the means of NRs between the sensors to controls (GFP). A two-tailed *t* test with a significance level of *P* = 0.05 was performed. Linear fit analysis was conducted to evaluate the correlation between OECT sensors’ NR and real concentration in clinical samples.

### MLP neural network

The neural network was constructed using the MLPClassifier module from the scikit-learn library. The architecture consisted of two hidden layers, each with 16 and 8 neurons, and used ReLU activation functions. The output layer consisted of three neurons with SoftMax activation, corresponding to the three disease stages. The model was trained using stochastic gradient descent with the Adam optimizer and a maximum of 2000 iterations. Performance was evaluated on a held-out 20% test set stratified by class.

## Supplementary Materials

### This PDF file includes:

Notes S1 and S2 Figs. S1 to S22 Legend for movie S1 References
