# Supplementary material for: Electronic profiling of neuronal extracellular vesicles enables early prediction of Parkinson’s disease
Source: Sci Adv. 2026 Sep 30;12(40):eaee4525. doi: 10.1126/sciadv.aee4525 (PMC13626060; doi:10.1126/sciadv.aee4525)
Supplement: Supplementary file 1 [file sciadv.aee4525_sm.pdf]

Science Advances
AAAS

Supplementary Materials for

Electronic profiling of neuronal extracellular vesicles enables early prediction of Parkinson's disease

Tianrui Chang *et al.*

Corresponding author: George K. Tofaris, george.tofaris@ndcn.ox.ac.uk; Keying Guo, keying.guo@gtiit.edu.cn; Sahika Inal, sahika.inal@kaust.edu.sa

Sci. Adv. **12**, eaee4525 (2026)
DOI: 10.1126/sciadv.aee4525

The PDF file includes:

Notes S1 and S2
Figs. S1 to S22
Legend for movie S1
References

Other Supplementary Material for this manuscript includes the following:

Movie S1

Supplementary Text

Supplementary Note 1. Interpretation of the OECT sensing mechanism

The variation in gate potential can be described by Equation 1, while the variation in gate capacitance is described by Equation 2. V_{gs} is distributed between the channel (Equation 3) and gate (Equation 4) EDLs according to their capacitance ratio. When target binding causes a change in C_g, the V_{gs} drop on the channel is modified as described by Equation 5, thereby altering the final measured I_{ds}.

$$\Delta\varphi_G = \frac{nQ_{tgt}}{\varepsilon_{tgt}\varepsilon_0} d_{tgt}$$

(1)

$$\Delta C_g = \frac{\Delta\varepsilon_r\varepsilon_0 A}{\Delta d}$$

(2)

$$V_{gs,drop}^{ch} = \frac{1}{1+\frac{C_{ch}}{C_g}} V_{gs}$$

(3)

$$V_{gs,drop}^{g} = \frac{1}{1+\frac{C_g}{C_{ch}}} V_{gs}$$

(4)

$$\Delta V_{gs,drop}^{ch} = V_{gs,drop,target}^{ch} - V_{gs,drop,blank}^{ch}$$

(5)

where $V_{gs,drop,target}^{ch} = f(C_{g,target})$ and $V_{gs,drop,blank}^{ch} = f(C_{g,blank})$.

n is the surface density of the target, Q_{tgt} is the charge of the target molecule, ε_0 is the dielectric permittivity of the vacuum, ε_{tgt} is the relative dielectric permittivity of the target and d_{tgt} is the target thickness. $\Delta\varepsilon_r$ is the variation of gate dielectric permittivity, A is the gate area, and Δd is the variation in gate biolayer thickness. $V_{gs,drop}^{ch}$ and $V_{gs,drop}^{g}$ represent the portion of V_{gs}that drop at the channel-electrolyte and gate-electrolyte interfaces, respectively. This drop before target binding is labelled as *blank*, while *target* after binding. $\Delta V_{gs,drop}^{ch}$represents the variation of gate voltage drop on the channel caused by the change in its capacitance originated by the target binding.

When the target binds to the gate electrode and its capacitance decreases ($\Delta C_g < 0$), the voltage drop across the capacitor, represented by the gate-electrolyte EDL, increases. As a consequence, the voltage drop across the channel-electrolyte EDL decreases, leading to a lower transmission of the gate voltage, i.e., lower V_{gs}^{eff} and lower doping.

Supplementary Note 2. Understanding Gate Capacitance Changes in the Clinical Sample

We performed zeta potential measurements for the commercial total α-Syn, α-Syn Agg samples, and a clinical sample (iRBD #1) that contains total α-Syn and α-Syn Agg. As shown in **Fig. S12**, the commercial α-Syn proteins exhibit a more negative zeta potential (-20 ~ −15 mV in 1% Triton/PBS), whereas the clinical sample has a weaker negative potential (−6 mV). Zeta potential reflects the effective surface charge that each suspended particle or macromolecule "shows" to the surrounding electrolyte; a higher-magnitude (more positive or more negative) value means stronger electrostatic repulsion between molecules and therefore better colloidal stability and less aggregation. Conversely, a lower-magnitude zeta potential indicates weaker electrostatic repulsion and a higher tendency for particles or proteins to come together and form clusters (*38, 39*). Based on the explanation above, the clinical sample (–6 mV) would be expected to aggregate or oligomerize more readily than the commercial sample (-20 ~ −15 mV), because a weaker net charge means weaker inter-particle repulsion and more self-association.

Electrochemical impedance spectroscopy (EIS) is sensitive to what kind of biolayer is created when the targets bind to the electrode surface, not just whether "something bound," but whether that the bound biolayer is compact and dielectric or swollen and ionic. Binding events change the dielectric constant, ion accessibility, thickness, and roughness of the biolayer at the electrode/electrolyte interface, and these changes show up as changes in interfacial capacitance-like terms (often modeled as a constant phase element, CPE) (*40, 41*). Two cases are commonly described in EIS immunosensors. One is a relatively uniform, densely packed antigen-antibody layer that forms a more blocking film that increases the effective distance between the electrode surface and mobile ions and tends to lower the apparent interfacial capacitance (or CPE magnitude) (*40*), which matches the decreased gate capacitance that we observed and reported in **Fig. S3A**. The other one describes a thicker, rough, water-rich, ion-permeable layer (i.e. when aggregated targets or bulky complexes accumulate) can increase the apparent interfacial capacitance, because they introduce a hydrated, polarizable matrix with counterions very close to the electrode. That behaves less like a dielectric coating and more like a hydrated, partially conductive "sponge," increasing the active area available for charge storage (*42*), which explains the increased gate capacitance shown in **Fig. S13B**.

To directly probe the origin of the OECT response, we performed in situ potential measurements of the gate, source, and drain electrodes referenced to a leakless Ag/AgCl electrode during OECT operation using a multi-channel potentiostat. The schematic of the experiment set up is now shown in **Fig. S14A**. This integrated setup allows us to quantitatively evaluate how the applied gate voltage is distributed across the gate/electrolyte and channel/electrolyte interfaces. We used an Agg antibody-functionalized sensor and tested with OECT before and after the clinical sample (includes α-Syn Agg) exposure. Five output curves were recorded for each case, and the 5th data was converted to the transfer curves and shown in **Fig. S14B**. The OECT showed a clear shift in transfer curve after incubation with the clinical sample (**Fig. S14B**). Simultaneously, we monitored the gate potential during device operation (five output curves). After the binding of the protein in the clinical sample to the gate electrode, the gate potential shifted to more negative values (from 260 mV to 253 mV) (**Fig. S14C**). We also observe that the applied voltage was no longer dropping

as much as it did for the "blank" case. This indicates that the gate electrode became less polarized after target binding. To further quantify this effect, we evaluated the relative voltage drop across the gate and channel interfaces and estimated the ratio between channel and gate capacitance (C_g/C_{ch}) by comparing the modulation amplitude of the source electrode (representing the channel side) and the gate electrode (**Fig. S14D**). After exposure to the clinical sample, the C_g/C_{ch} increased, indicating an increase in C_g relative to the channel capacitance. This observation is consistent with the formation of a thicker, hydrated, and ion-permeable biolayer upon binding of clinical samples. Such a layer increases the effective interfacial capacitance and reduces the voltage drop at the gate interface, thereby increasing the effective gate voltage transmitted to the channel. Importantly, although the surface potential decreases after target binding (as evidenced by the negative shift in gate potential), its effect is outweighed by the increase in C_g, which enhances the effective gating of the channel. As a result, the net transistor response dominated by the capacitance effect, leading to an increase in channel current (**Fig. S14B**).

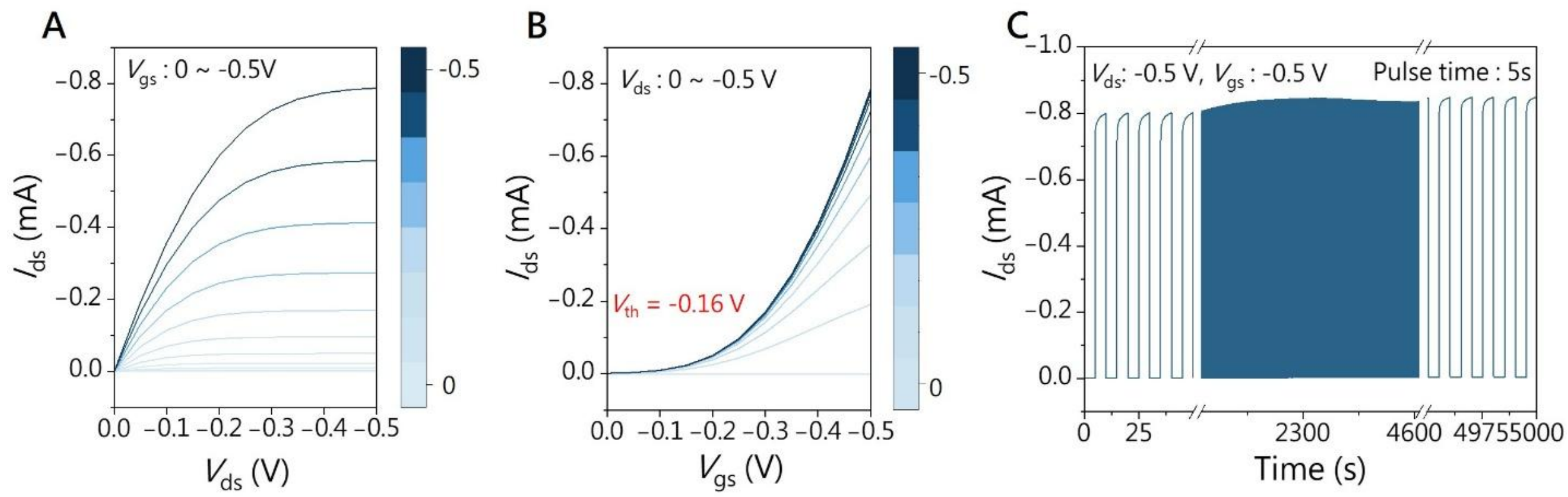

Fig. S1. OECT Characteristics. (A) An exemplary output curve and **(B)** transfer curve of the p-type OECT gated by a gold coil in 40 mM PB. The colored bars represent correlated V_{ds} **(A)** and V_{gs} **(B)**. **(C)** The channel current at V_{ds} = -0.5 V was monitored for 5000 seconds under continuous V_{gs} = 0 V and -0.5 V pulses applied for 5 s each.

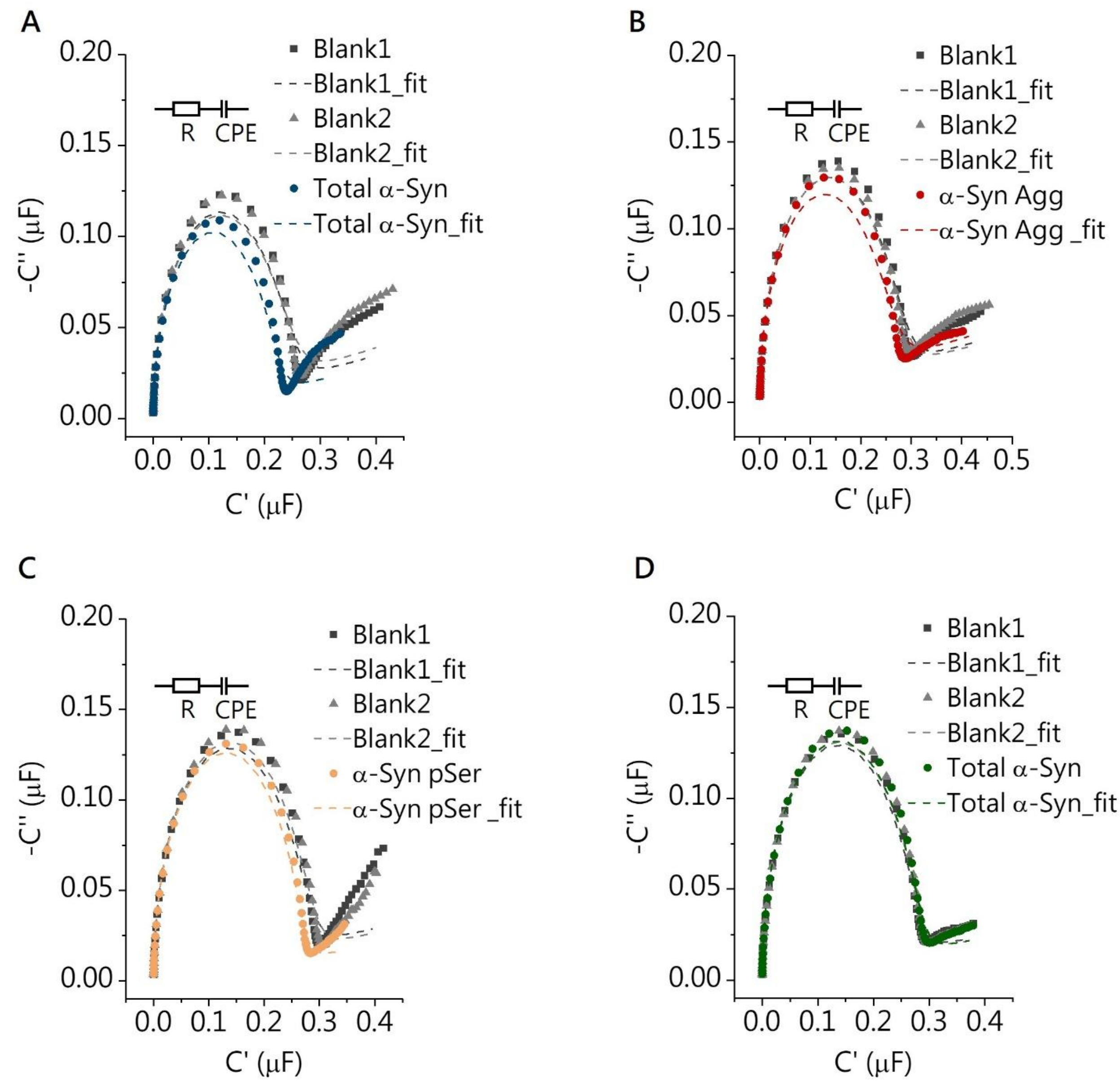

Fig. S2. Cole-Cole plots with RCPE circuit fitting of (A) total α-Syn, **(B)** α-Syn Agg, **(C)** α-Syn pSer, and **(D)** GFP control sensors before (Blank 1 and Blank 2) and after exposure to their respective commercial α-Syn targets.

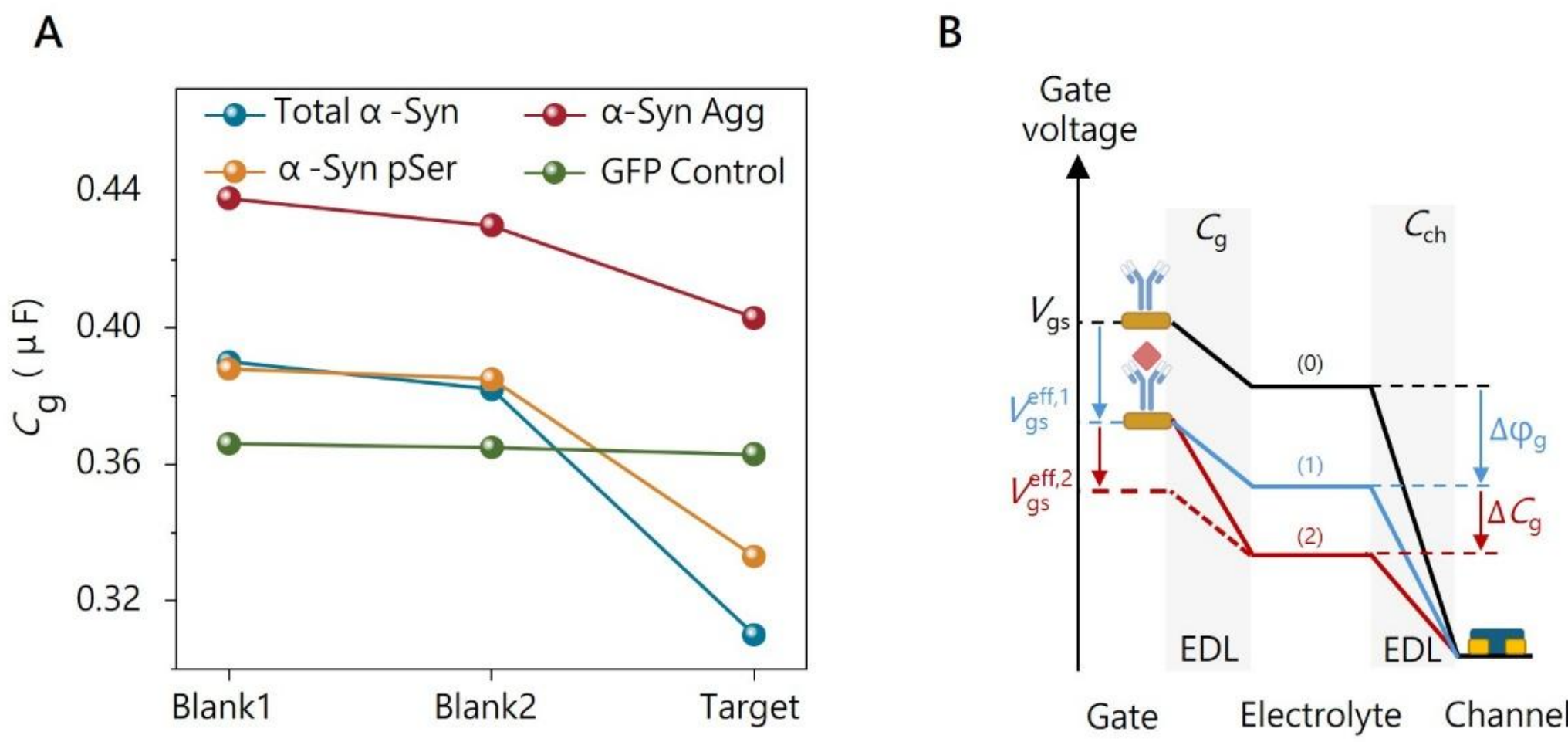

Fig. S3. **(A)** Gate capacitance (C_g) extracted by fitting the EIS data to a RCPE circuit, showing reductions in interfacial capacitance upon target binding. **(B)** Schematic illustrating how decreases in gate surface potential (φ_g) and capacitance (C_g) combine to modulate the effective gate voltage V_{gs}^{eff} in a p-type OECT. Condition (0) represents the OECT before target binding, condition (1) isolates the effect of a reduced φ_g following target binding, and condition (2) includes the effect of C_g decrease, leading to the overall change in the effective gate voltage $V_{gs}^{eff,2}$.

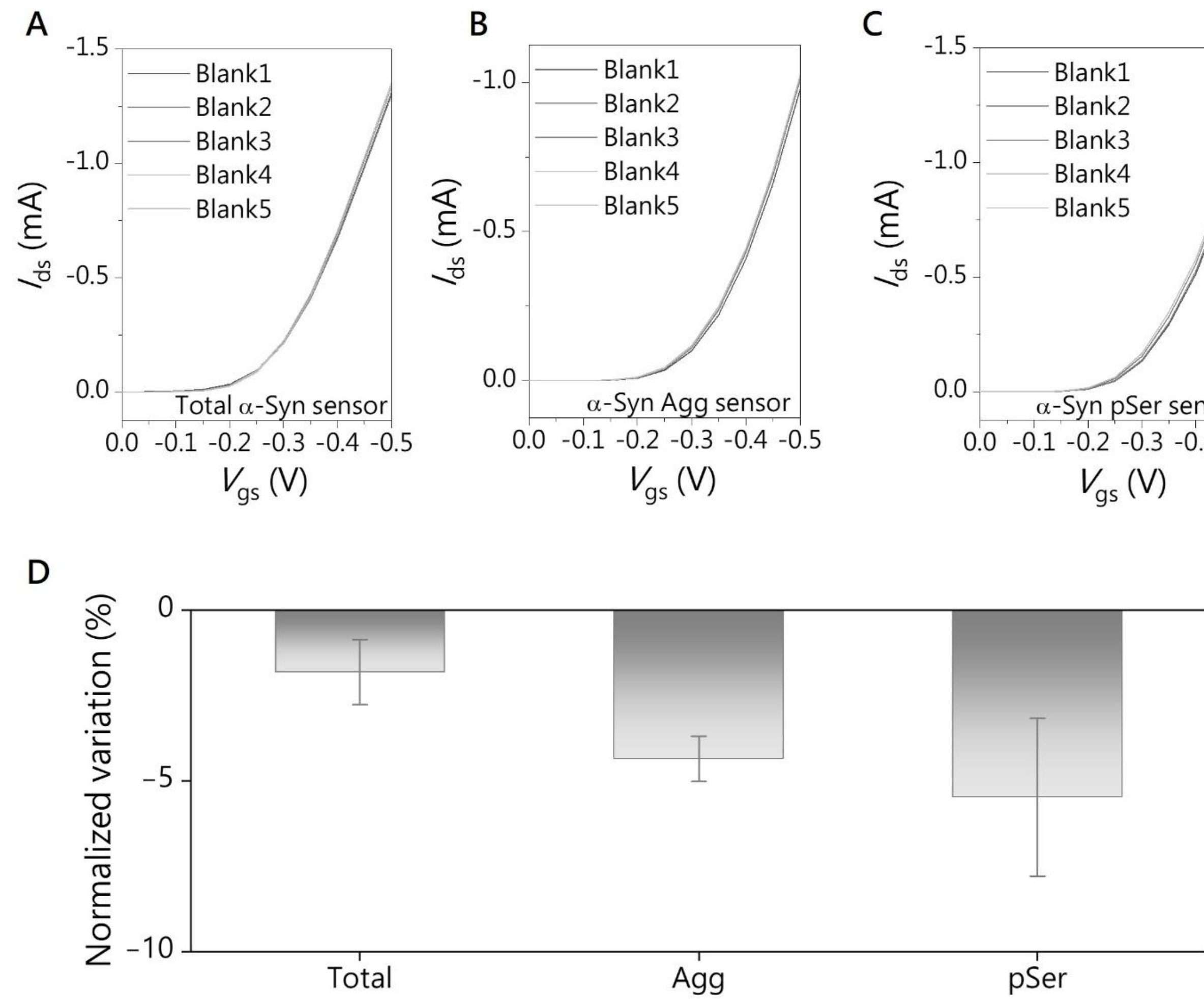

Fig. S4. Blank stability tests of α-Syn sensors. Transfer curves from OECT sensors after successive incubations with multiple blank samples (1% Triton) to investigate possible drift: **(A)** Total α-Syn, **(B)** α-Syn Agg, and **(C)** α-Syn pSer sensors. Transfer curves shown were recorded at V_{ds} = -0.5 V. **(D)** Normalized variation of the blank stability tests of α-Syn sensors.

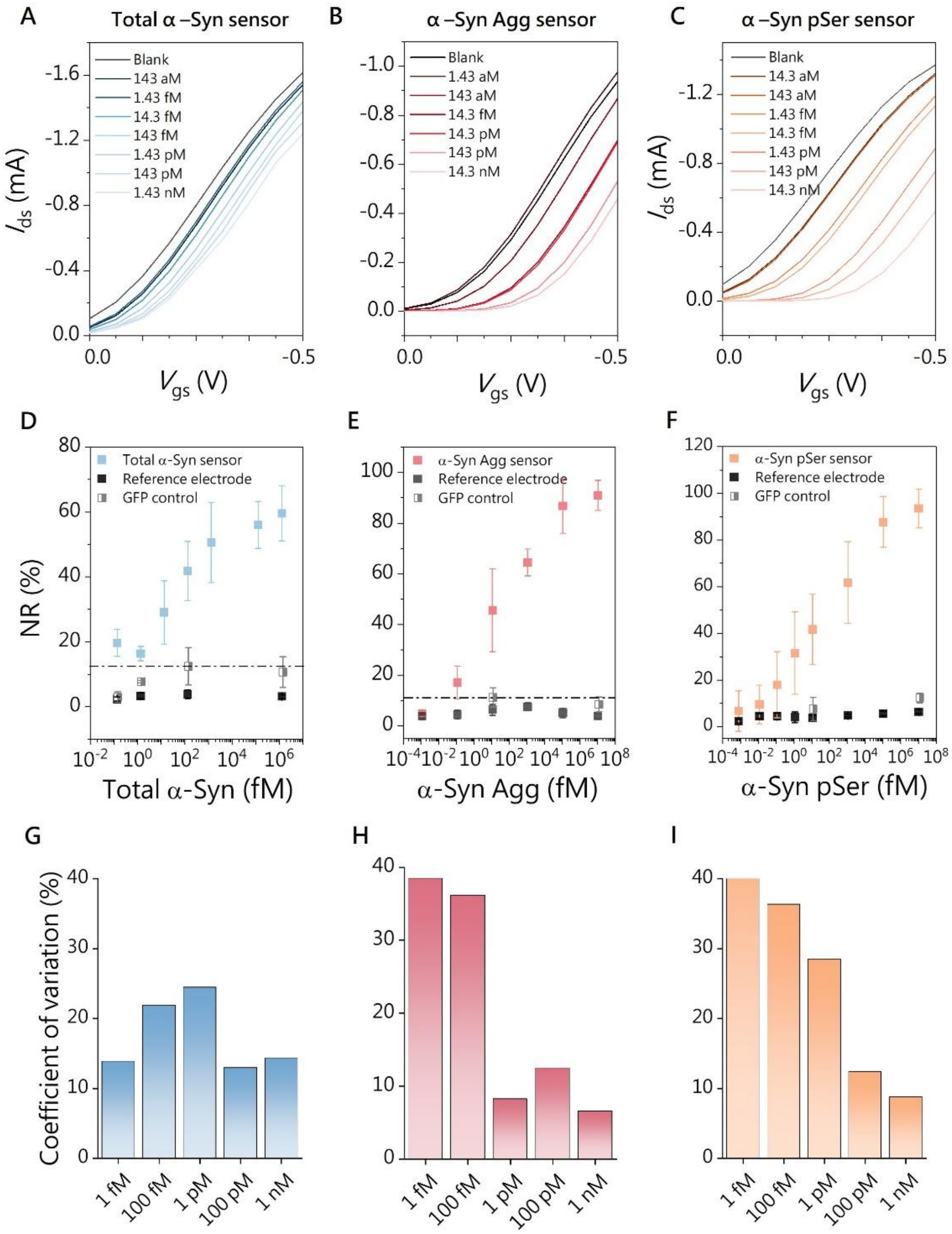

Fig. S5. The sensing performance of commercial α-Syn target proteins in 1% Triton. **(A-C)** The transfer curves of each sensor to increasing concentrations of their respective targets: total α-Syn, α-Syn Agg, and α-Syn pSer. **(D-F)** Normalized current response of sensors for total α-Syn, α-Syn Agg, and α-Syn pSer. The black symbols are data from the gold coil reference electrode gated channels. The grey symbols are the response of GFP control electrode gated channels when they are exposed to some selected samples at the same time as the sensors. **(G-I)** Quantitative analysis of device-to-device reproducibility. Coefficient of variation (CV) of the response of three independent devices at each target concentration: **(G)** total α-Syn, **(H)** α-Syn Agg, and **(I)** α-Syn pSer sensors. CV values were obtained as the ratio of the standard deviation to the mean NR. Higher variability is observed at lower concentrations due to reduced signal-to-noise ratio.

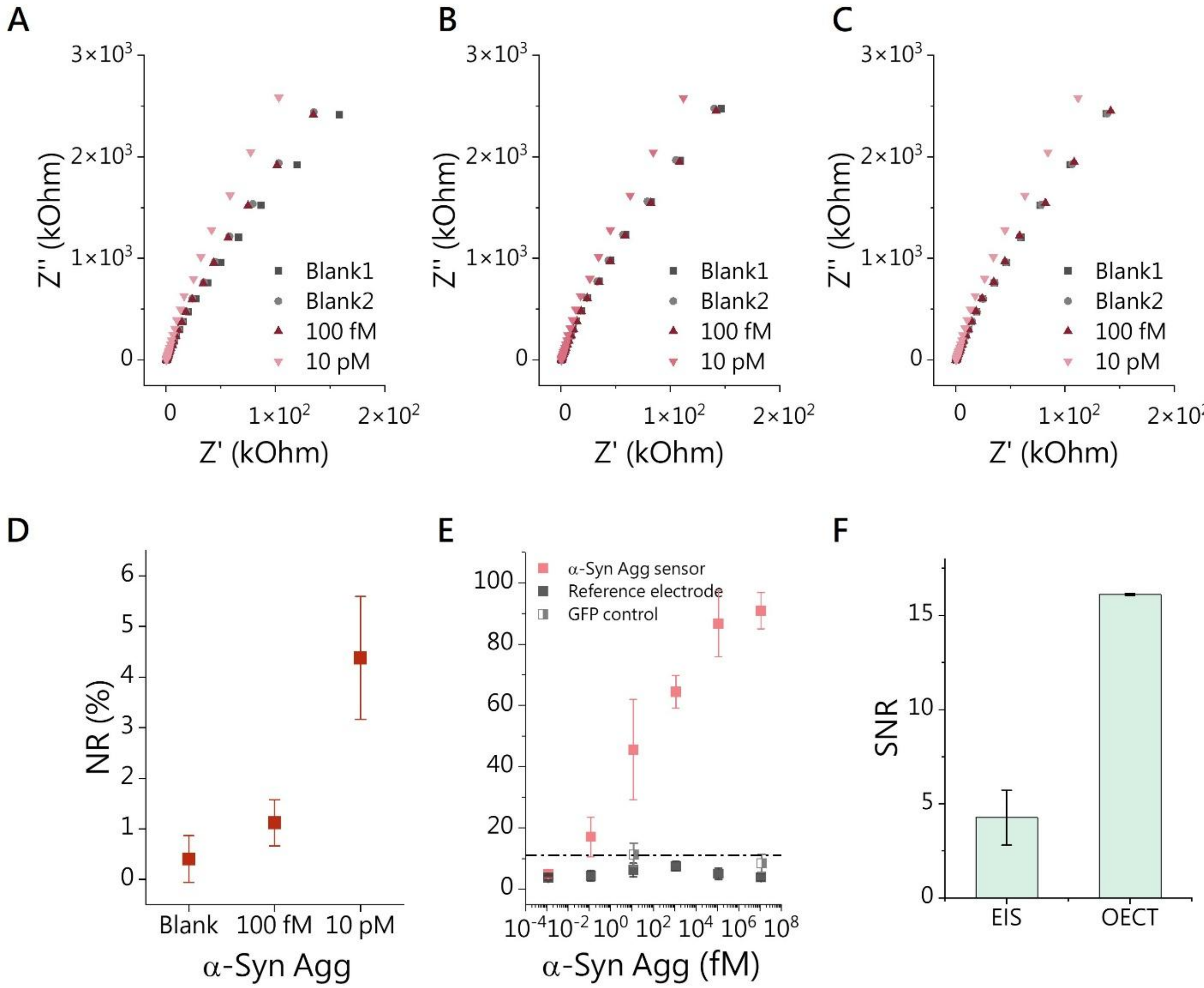

Fig. S6. Comparison of signal-to-noise ratio (SNR) between EIS and OECT for α-Syn Agg detection. (A–C) Nyquist plots of biofunctionalized electrodes for α-Syn Agg detection measured after sequential incubation steps: Blank1 (1% Triton), Blank2 (1% Triton), 100 fM α-Syn Agg (in 1% Triton), and 10 pM α-Syn Agg (in 1% Triton). Measurements were performed over a frequency range of 0.1 Hz to 1 MHz in 40 mM PB (three-electrode configuration) at open circuit conditions. **(D)** Normalized response of the electrodes calculated using C_g as the output extracted using an R–CPE circuit fit to the Nyquist spectra. **(E)** Calibration curve of α-Syn Agg OECT sensors was shown in **Fig. S5D**. **(F)** Comparison of signal-to-noise ratio (SNR) of the electrode and OECT based sensors. The SNR of the electrode sensors was calculated from the normalized capacitance change at 10 pM relative to blank variation, while the SNR for OECT was derived from the normalized current change at 10 pM relative to blank variation shown in **Fig. S4D**. Error bars represent standard deviation (n = 3 independent electrodes).

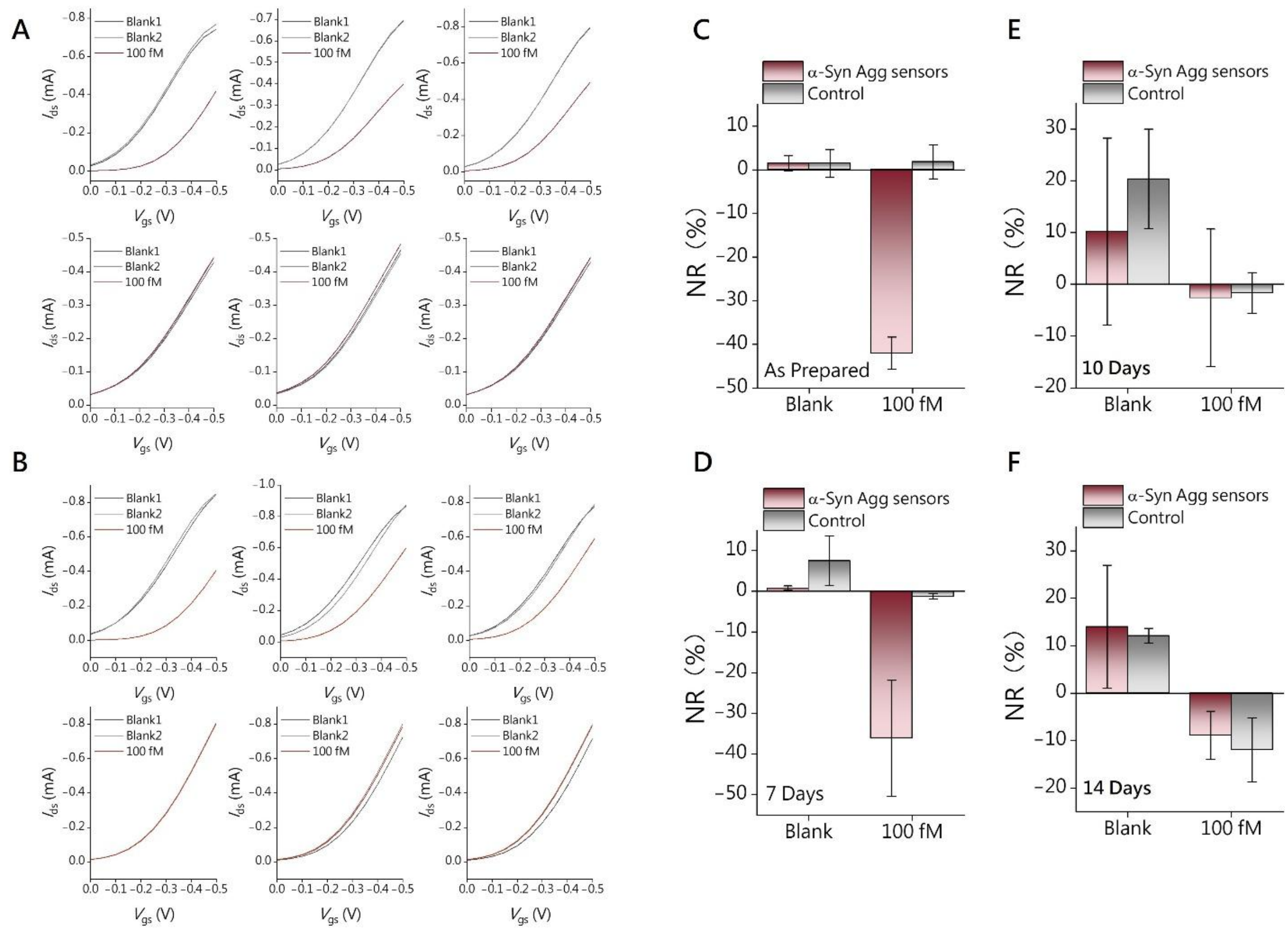

Fig. S7. Storage stability of α-Syn Agg antibody-functionalized gate electrodes. (A, B) Representative OECT transfer curves of α-Syn Agg sensors and GFP control electrodes measured after incubation with blank solution (1% Triton, Blank1 and Blank2) and 100 fM α-Syn Agg (in 1% Triton). Top rows are transfer curves from Agg sensors, and bottom rows are transfer curves from GFP controls. **(A)** As-prepared sensors and **(B)** sensors stored for 7 days at 4 °C in 1× PBS. **(C, D)** Corresponding normalized responses (NR) of α-Syn Agg sensors and GFP controls for freshly prepared electrodes **(C)** and after 7 days of storage **(D)**. **(E, F)** Normalized responses (NR) after extended storage for **(E)** 10 days and **(F)** 14 days. Normalized responses were calculated at $V_{ds} = V_{gs} = -0.5$ V. Error bars represent standard deviation (n = 3 independent electrodes).

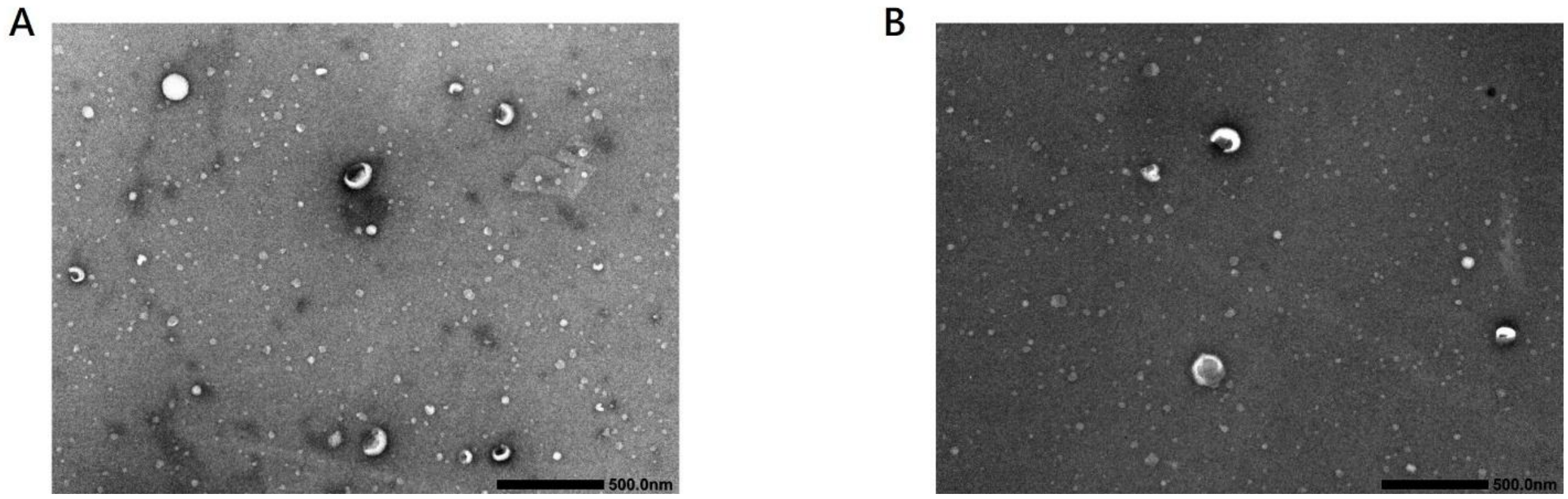

Fig. S8. TEM image of typical captured and eluted L1CAM+ EVs. The images suggest the double-membrane organization structure and a size of ~100 nm. Negative staining was performed before TEM imaging.

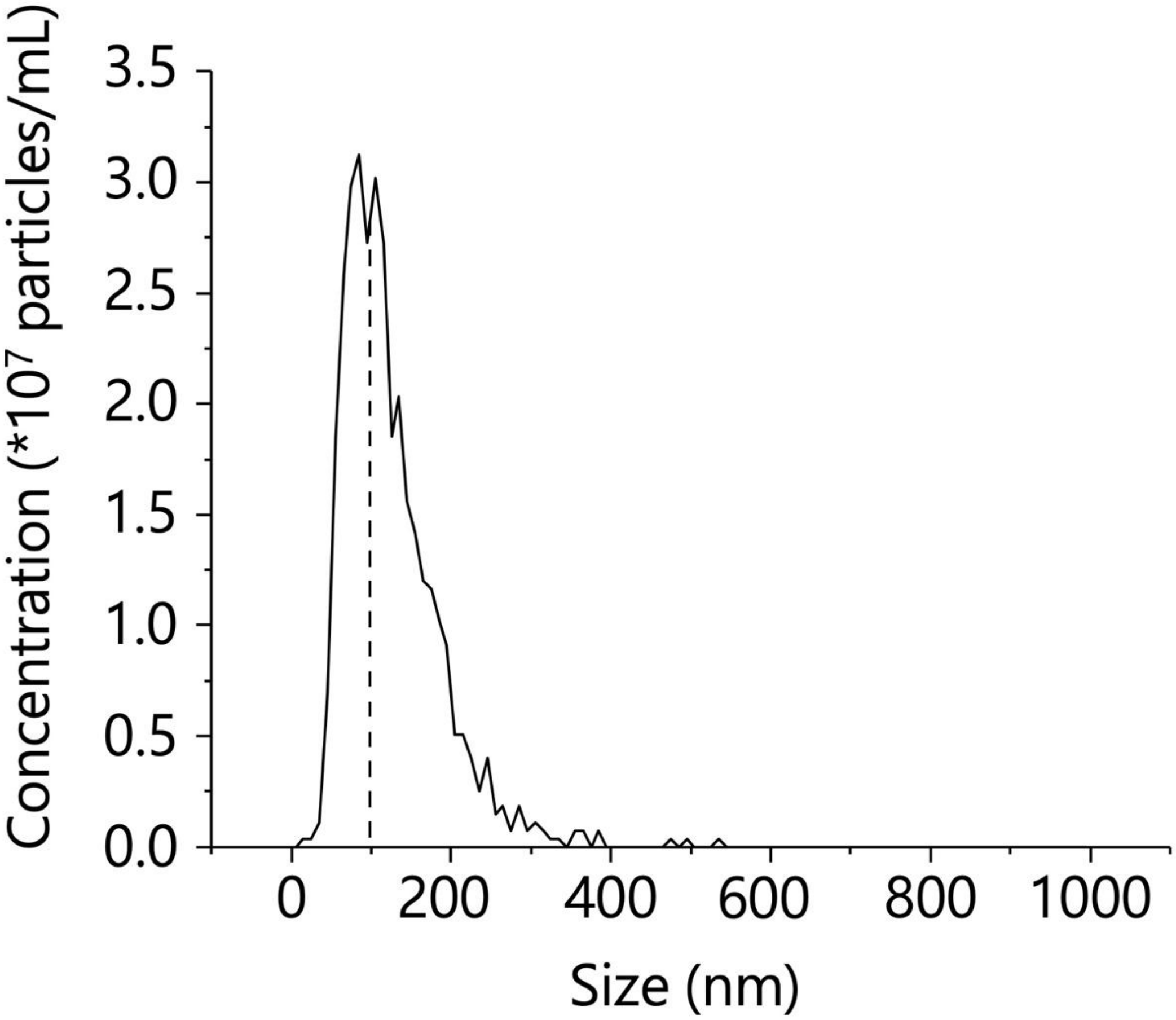

Fig. S9. A representative size distribution of captured and eluted L1CAM$^+$ EVs measured using Nanoparticle Tracking Analysis (NTA).

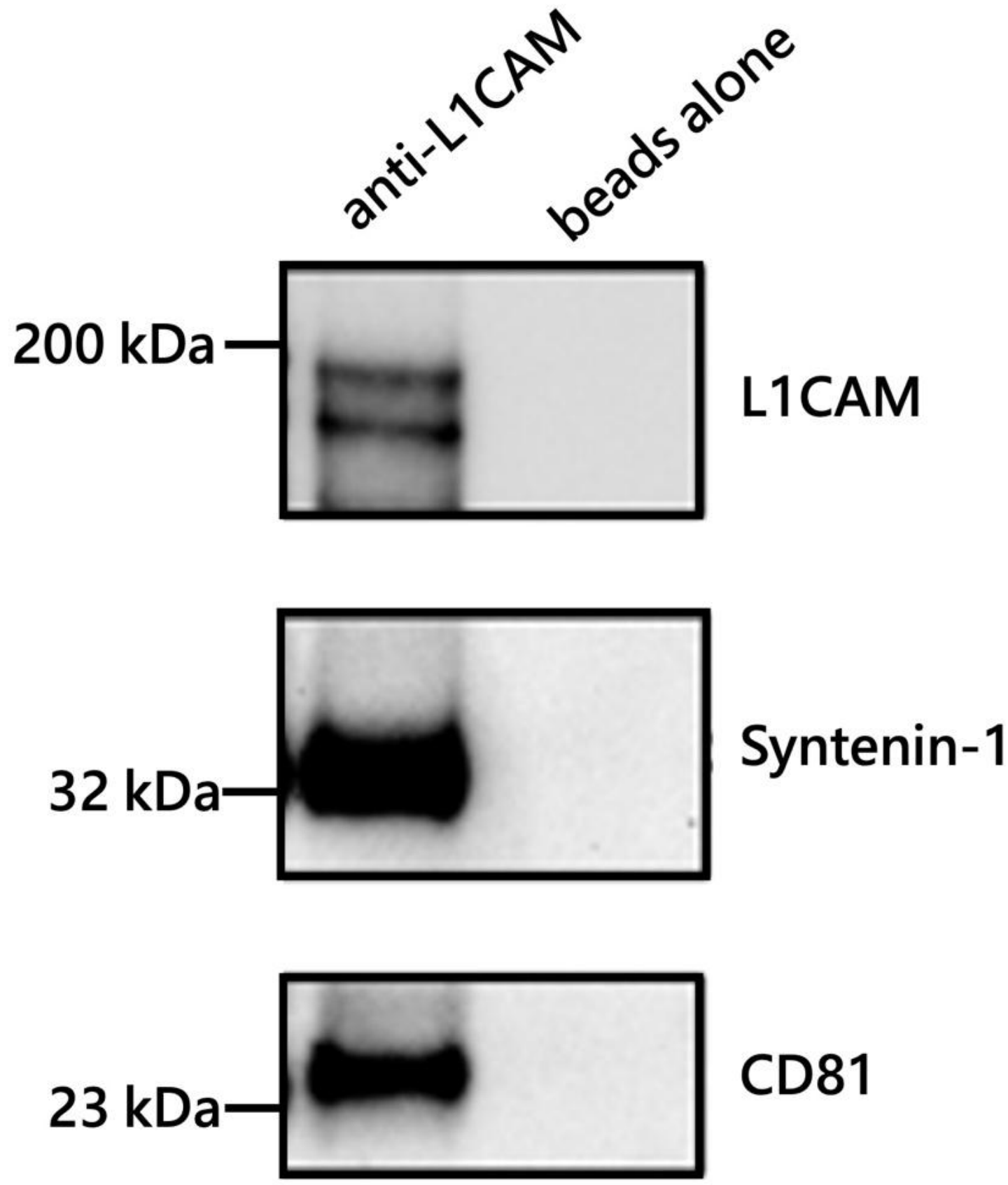

Fig. S10. Confirmation of EV isolation from serum. Immunocapture using anti-L1CAM from EV-enriched fractions obtained by size exclusion chromatography, followed by immunoblotting. Beads alone without antibody conjugation served as a negative control.

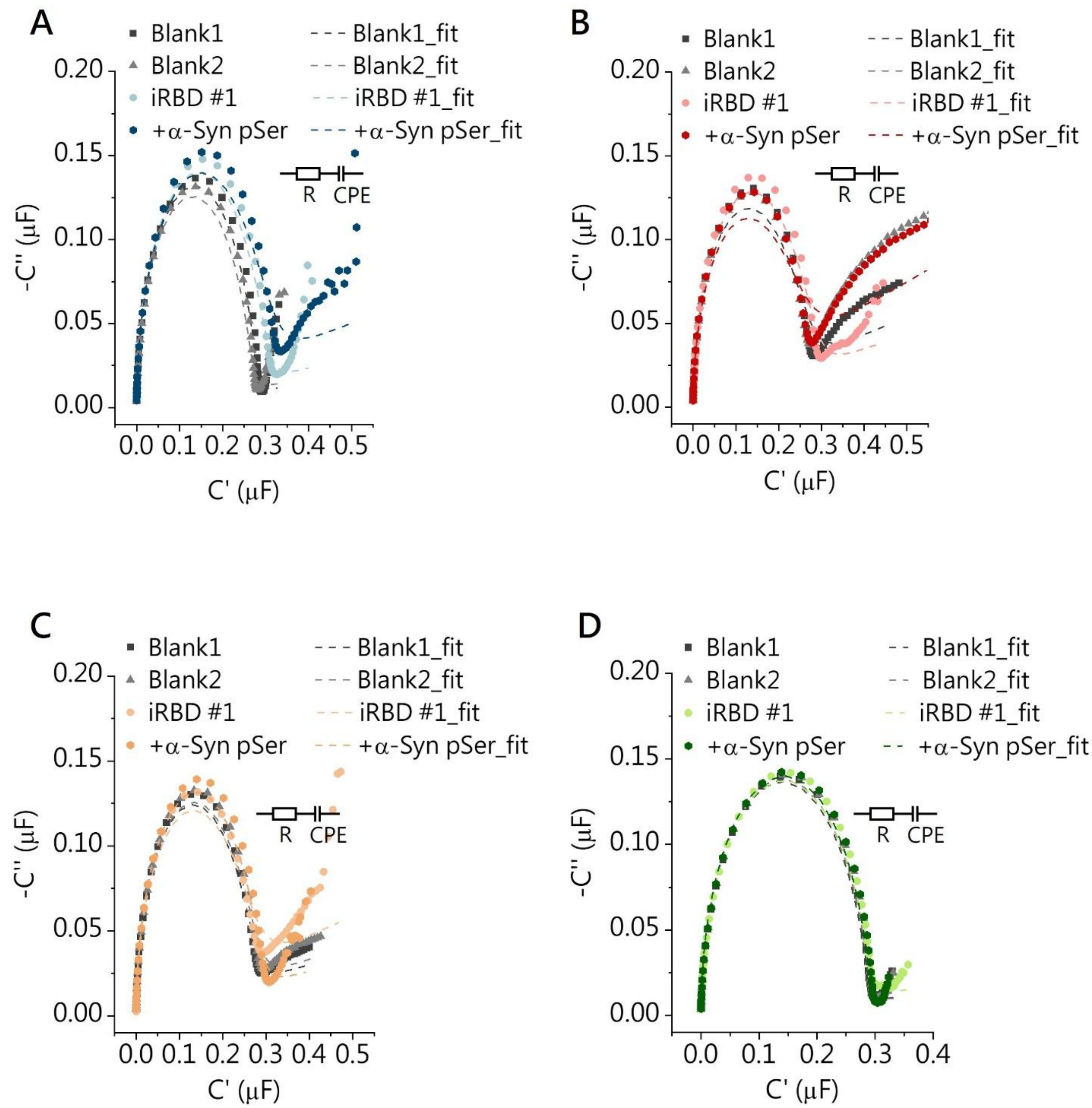

Fig. S11. Cole-Cole plots with RCPE circuit fitting of (A) total α-Syn, **(B)** α-Syn Agg, **(C)** α-Syn pSer, and **(D)** GFP control sensors. Each plot shows two baseline measurements (Blank 1 and Blank 2) followed by the response to a representative clinical iRBD sample and to the α-Syn pSer-spiked clinical iRBD sample.

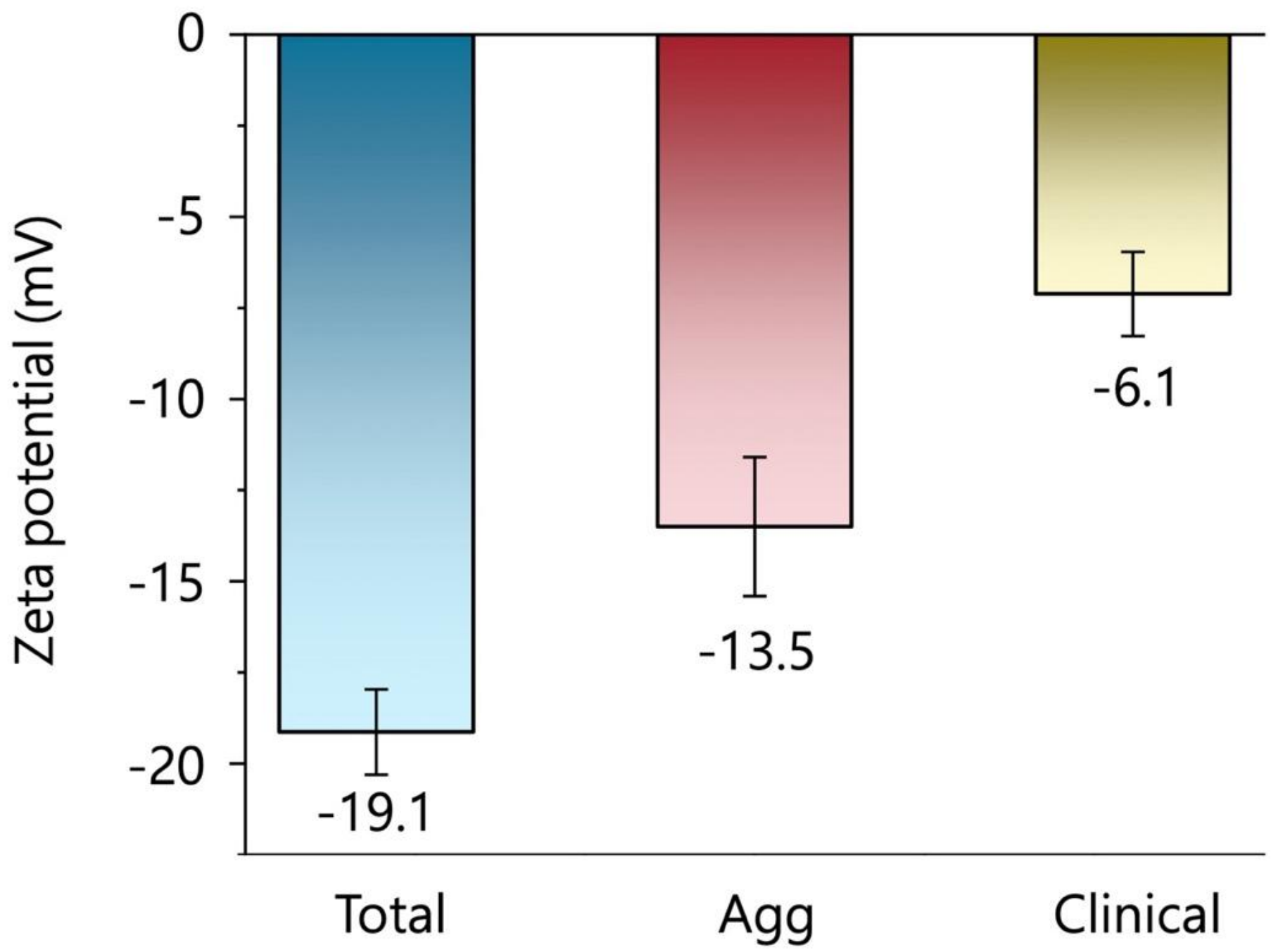

Fig. S12. Zeta potential of commercial total α-Syn protein, commercial α-Syn Agg protein, and a clinical sample (iRBD #1) in 1% Triton (in 1× PBS).

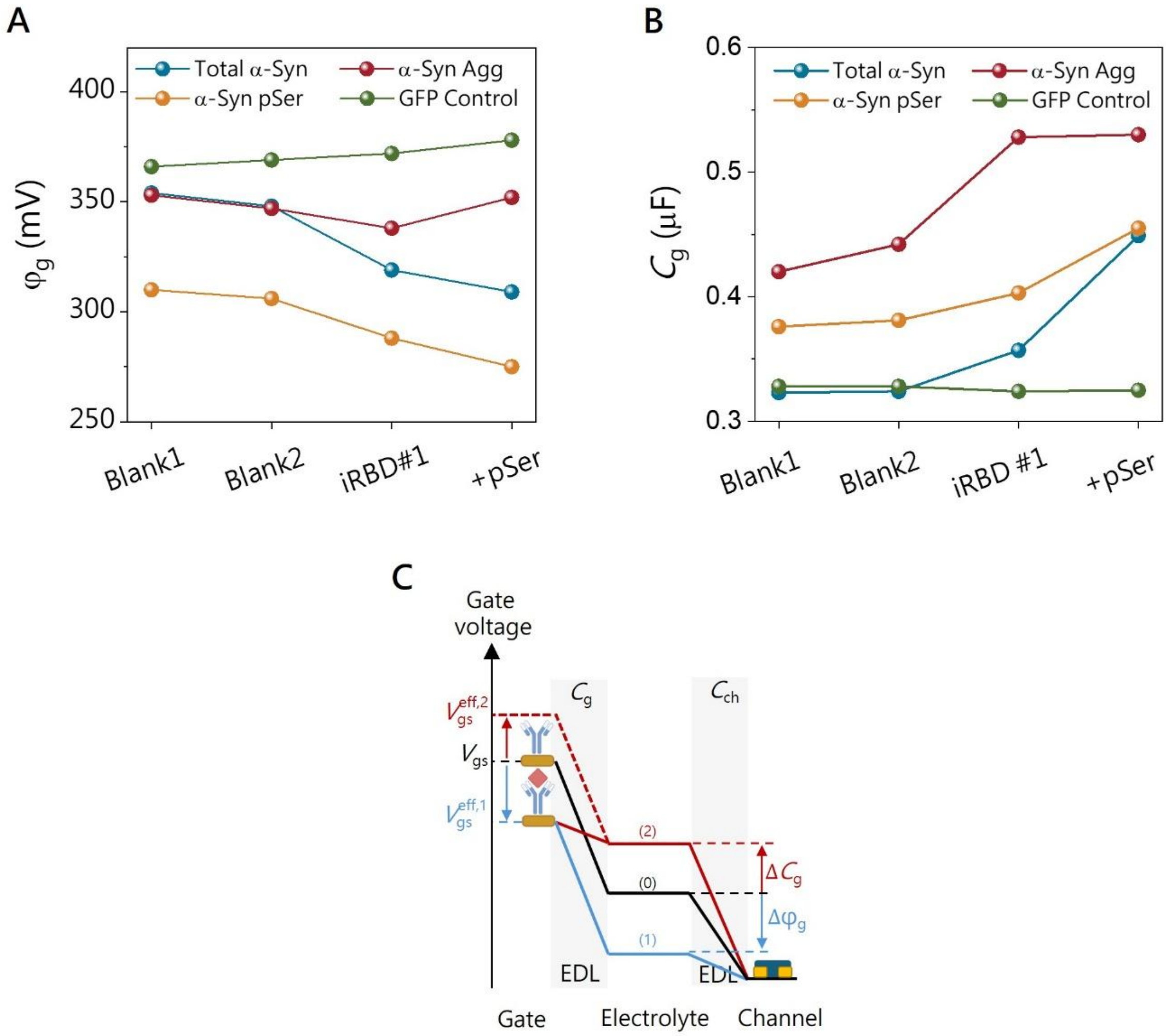

Fig. S13. EIS characterization and sensing schematic of sensors exposed to clinical samples. **(A)** The changes in the surface potential of the gate electrode. **(B)** The changes of C_g fitted from the RCPE circuit. **(C)** The schematic describes how the combined change, i.e., gate φ_g decrease and C_g increase, affect the voltage drops at the channel.

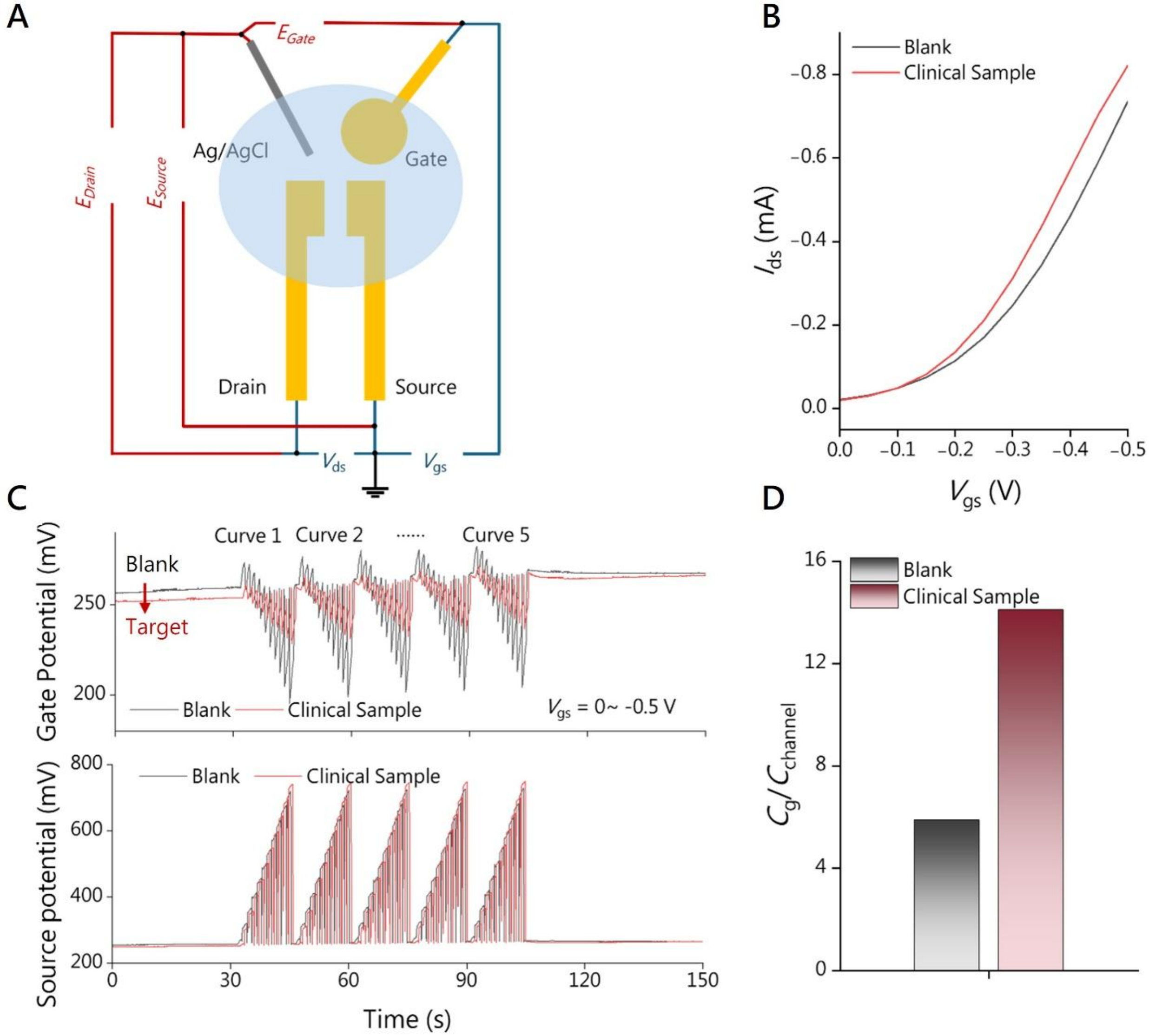

Fig. S14. Experimental validation of mixed potential and capacitance-dominated gating in clinical samples. (A) Schematic of the experimental set up. **(B)** OECT transfer curves of an α-Syn Agg sensor before and after incubation with a representative clinical sample. **(C)** Measured gate electrode potential (top) and source electrode potential (bottom) (vs. leakless Ag/AgCl) during OECT operation before (black) and after (red) clinical sample exposure. Five consecutive output curve measurements were recorded for each condition. **(D)** Ratio of gate-to-channel capacitance estimated from the relative source and gate potentials.

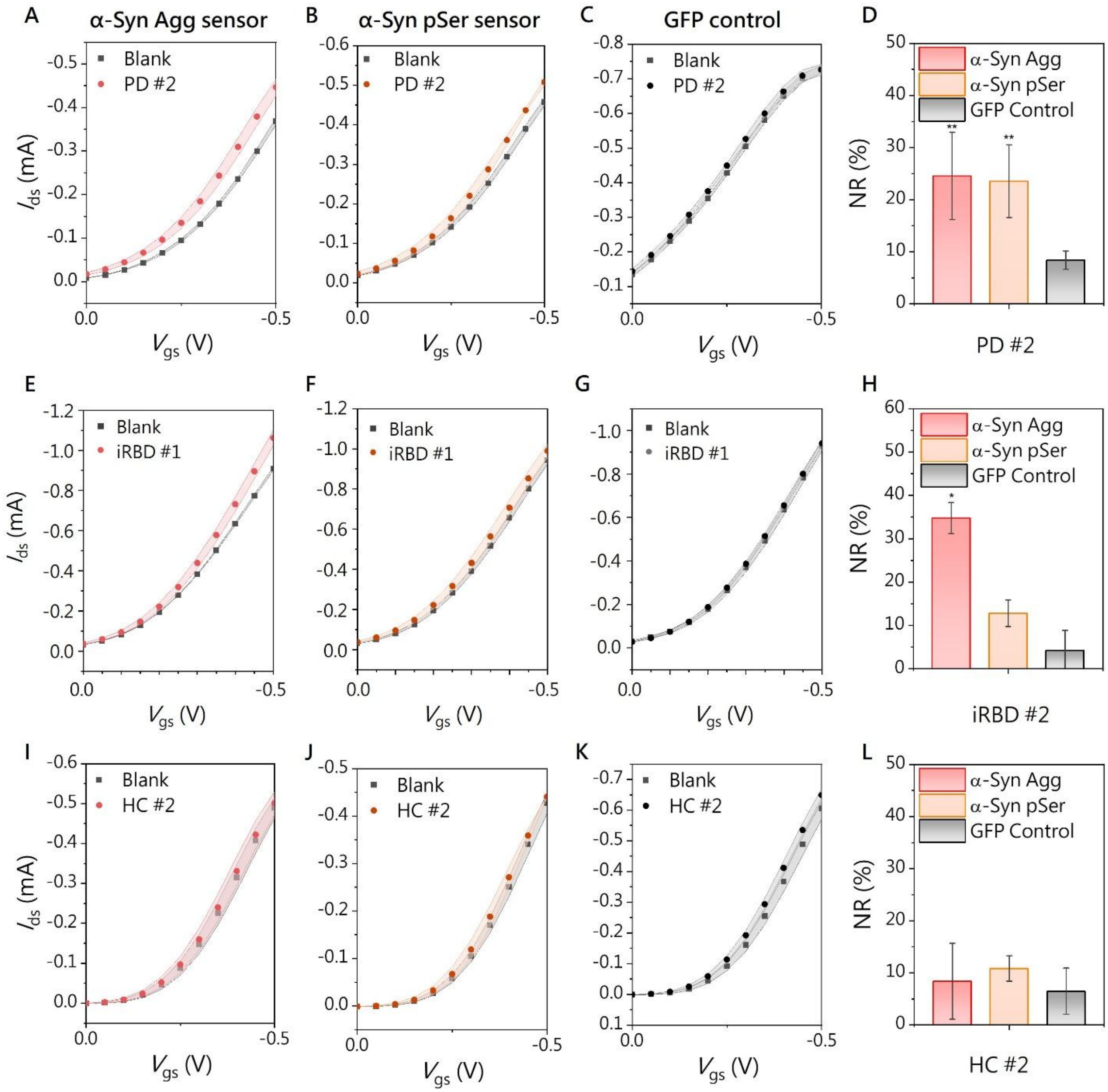

Fig. S15. Sensor response to clinical samples (group 2). (A-D) PD sample, **(E-H)** iRBD sample, **(I-L)** HC sample. The error bars and bands were calculated using data from three sensors (3 independent gate electrode arrays) incubated with the same sample. The red and orange bars indicate responses from α-Syn Agg and α-Syn pSer sensors, respectively, while the grey bars represent the response from controls. Transfer curves shown are under a V_{ds} = -0.5 V. NRs are calculated at $V_{ds} = V_{gs}$ = -0.5 V.

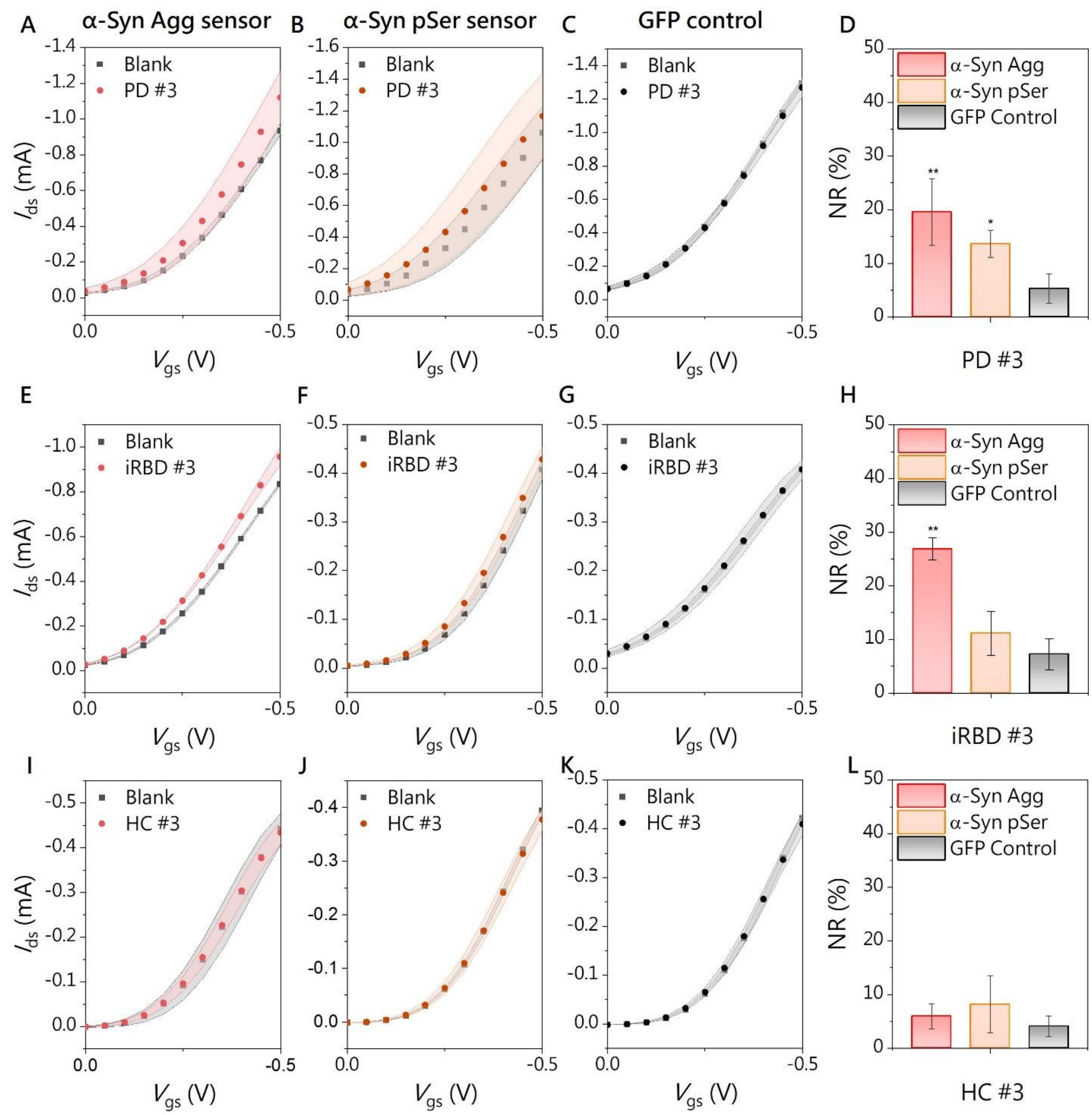

Fig. S16. Sensor response to clinical samples (group 3). (A-D) PD sample, **(E-H)** iRBD sample, **(I-L)** HC sample. The error bars and bands were calculated using data from three sensors (3 independent gate electrode arrays) incubated with the same sample. The red and orange bars indicate responses from α-Syn Agg and α-Syn pSer sensors, respectively, while the grey bars represent the response from controls. Transfer curves shown are under a V_{ds} = -0.5 V. NRs are calculated at V_{ds} = V_{gs} = -0.5 V.

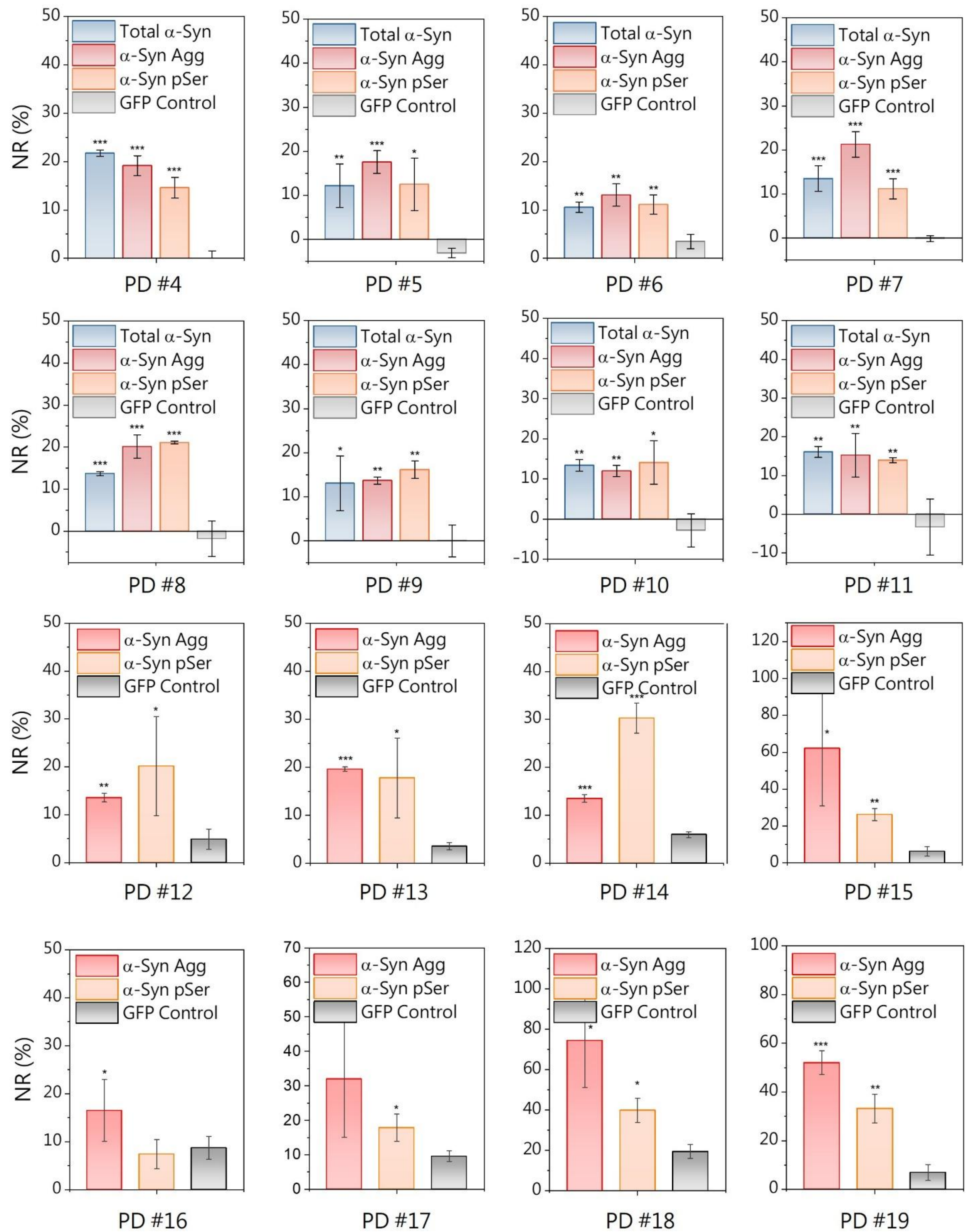

Fig. S17. Blind test results that identified PD clinical samples. NRs are calculated at $V_{ds} = V_{gs} = -0.5$ V.

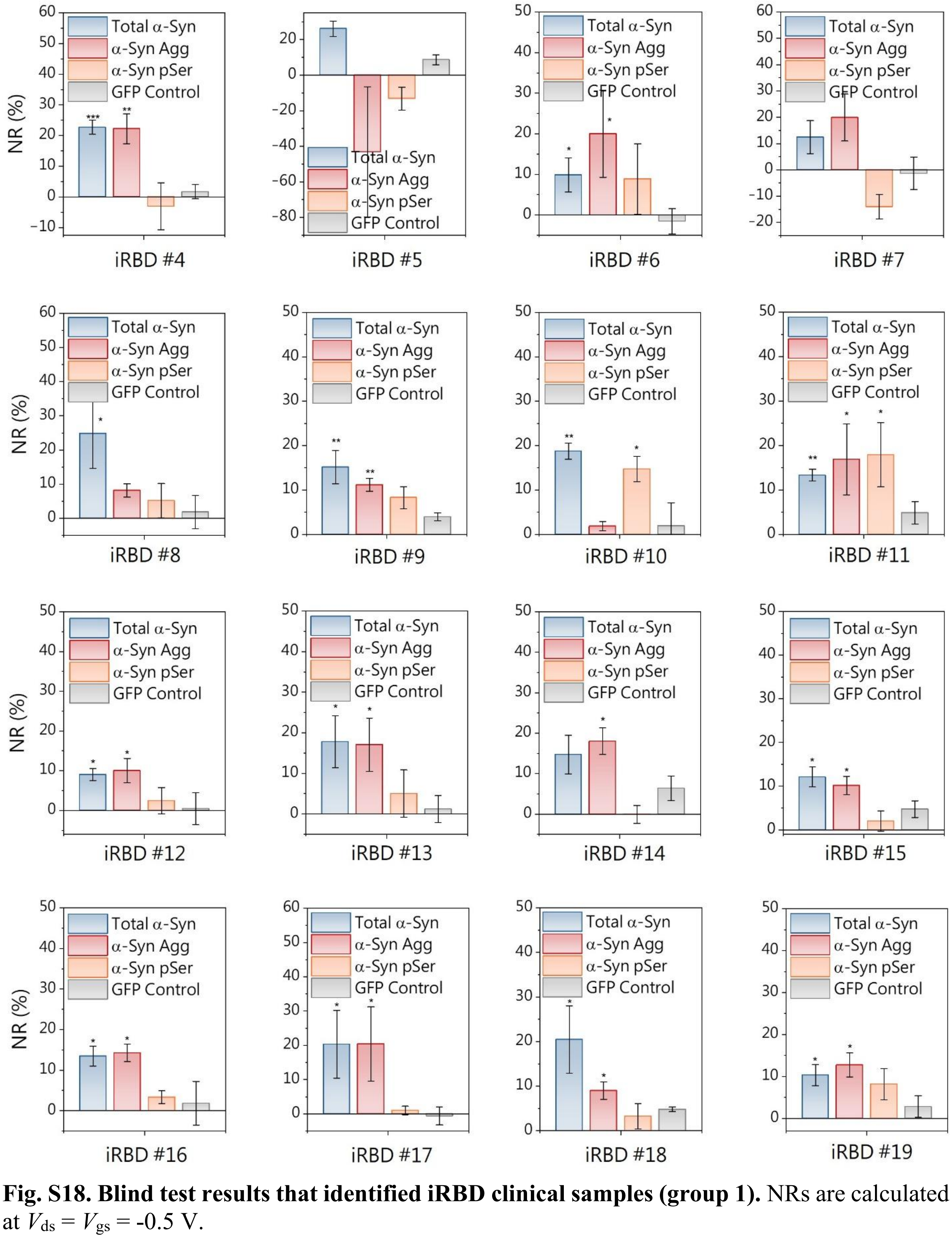

Fig. S18. Blind test results that identified iRBD clinical samples (group 1). NRs are calculated at $V_{ds} = V_{gs} = -0.5$ V.

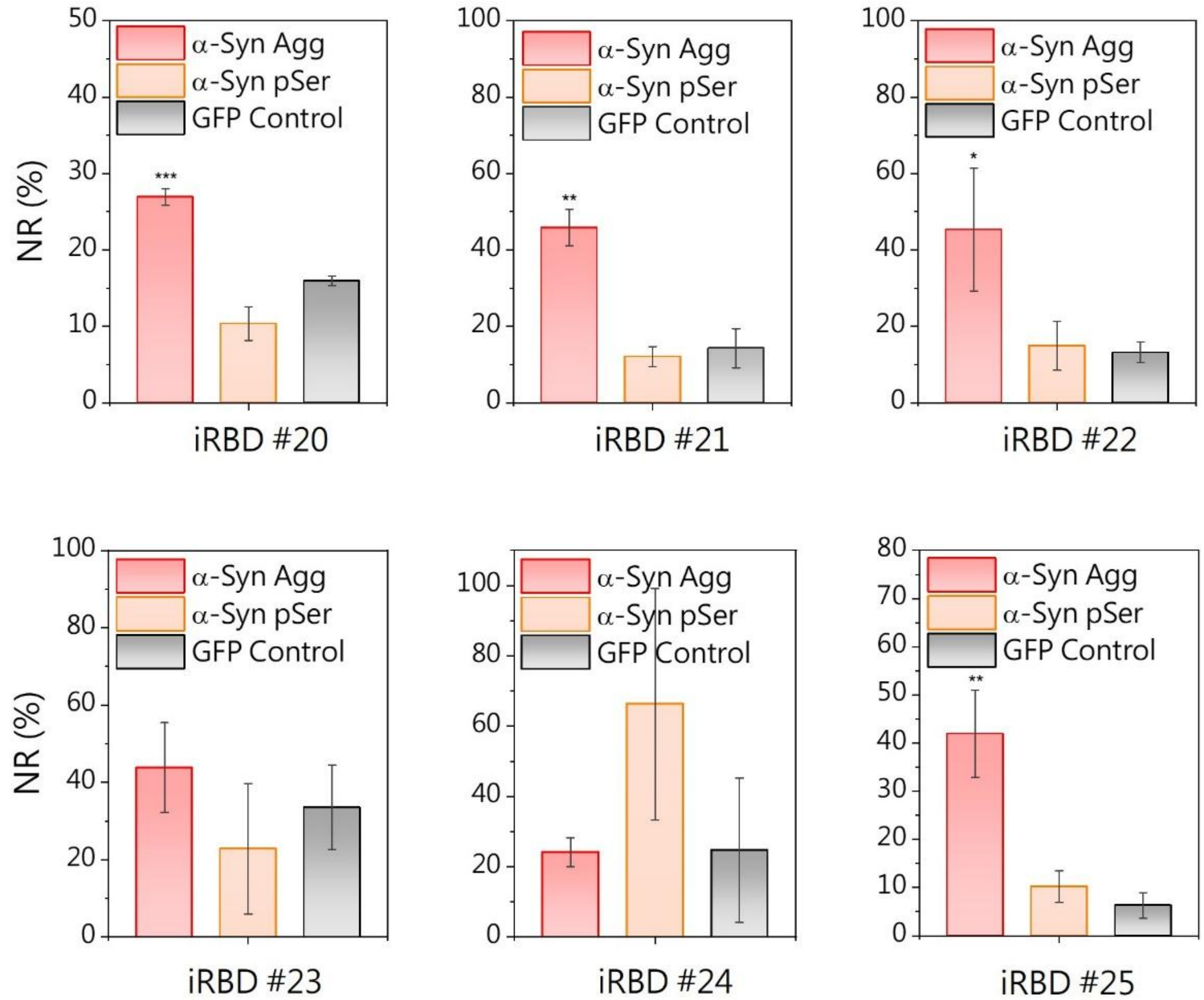

Fig. S19. Blind test results that identified iRBD clinical samples (group 2). NRs are calculated at $V_{ds} = V_{gs} = -0.5$ V.

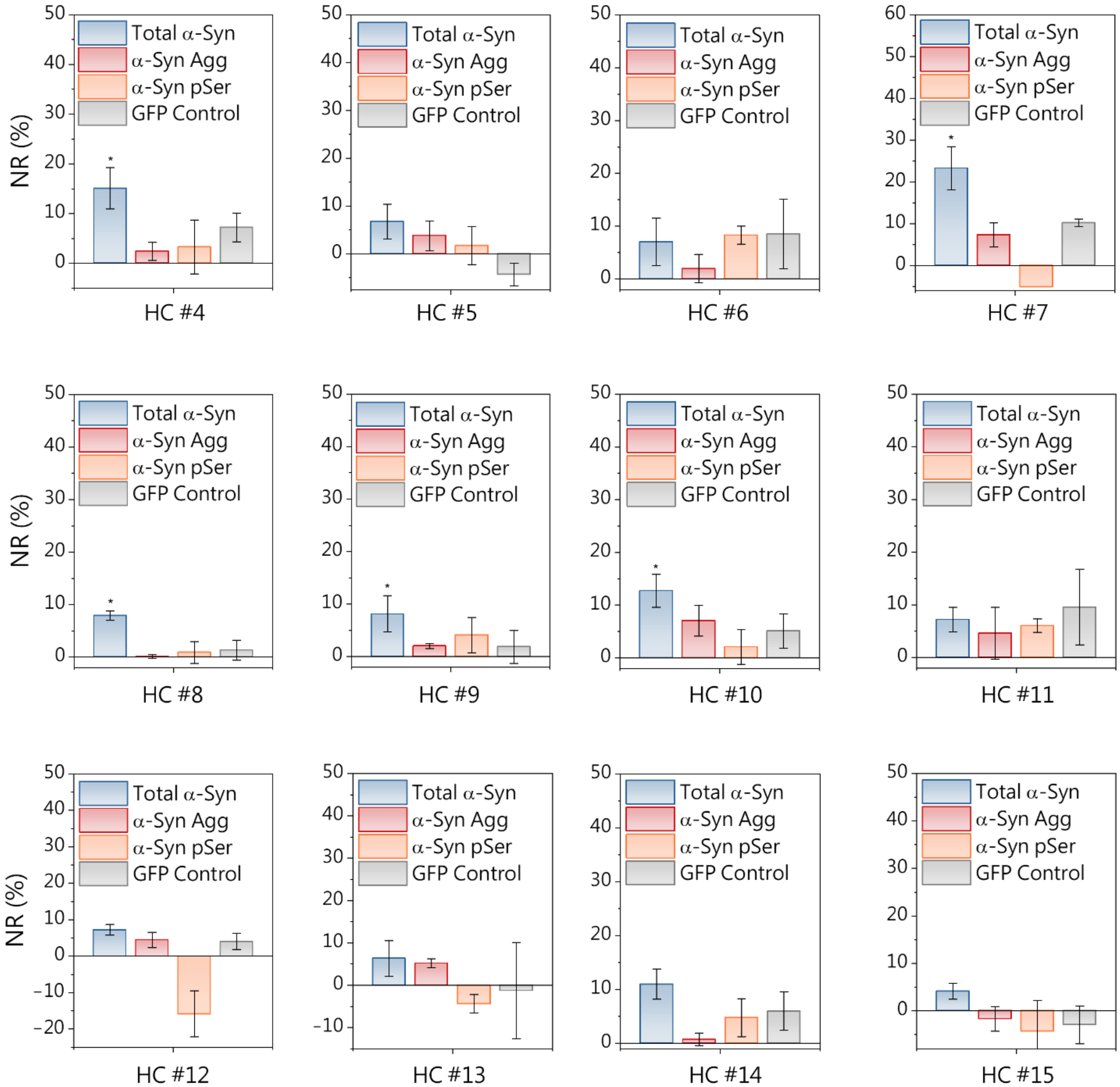

Fig. S20. Blind test results that identified HC clinical samples (group 1). NRs are calculated at $V_{ds} = V_{gs} = -0.5$ V.

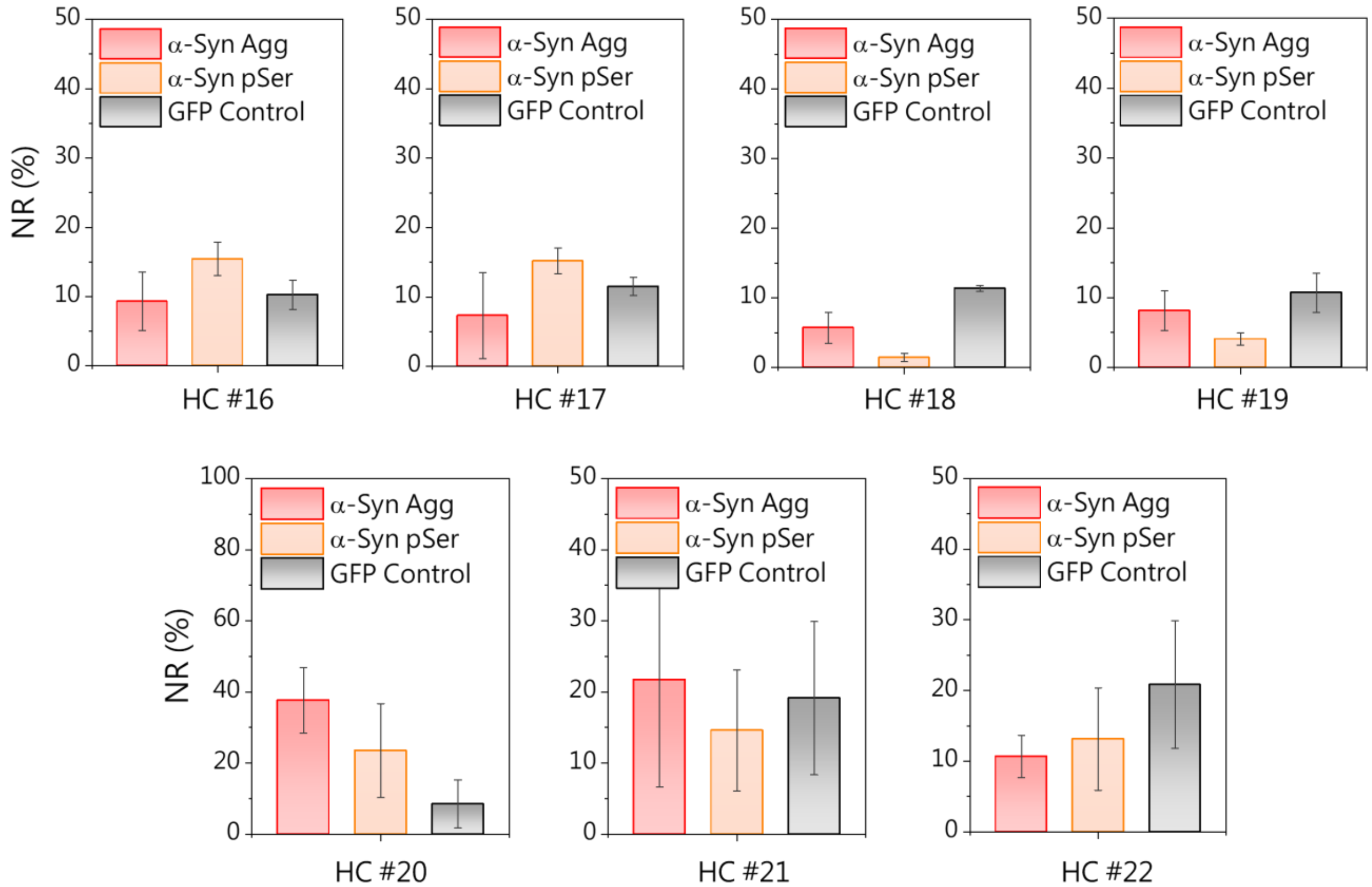

Fig. S21. Blind test results that identified HC clinical samples (group 2). NRs are calculated at $V_{ds} = V_{gs} = -0.5$ V.

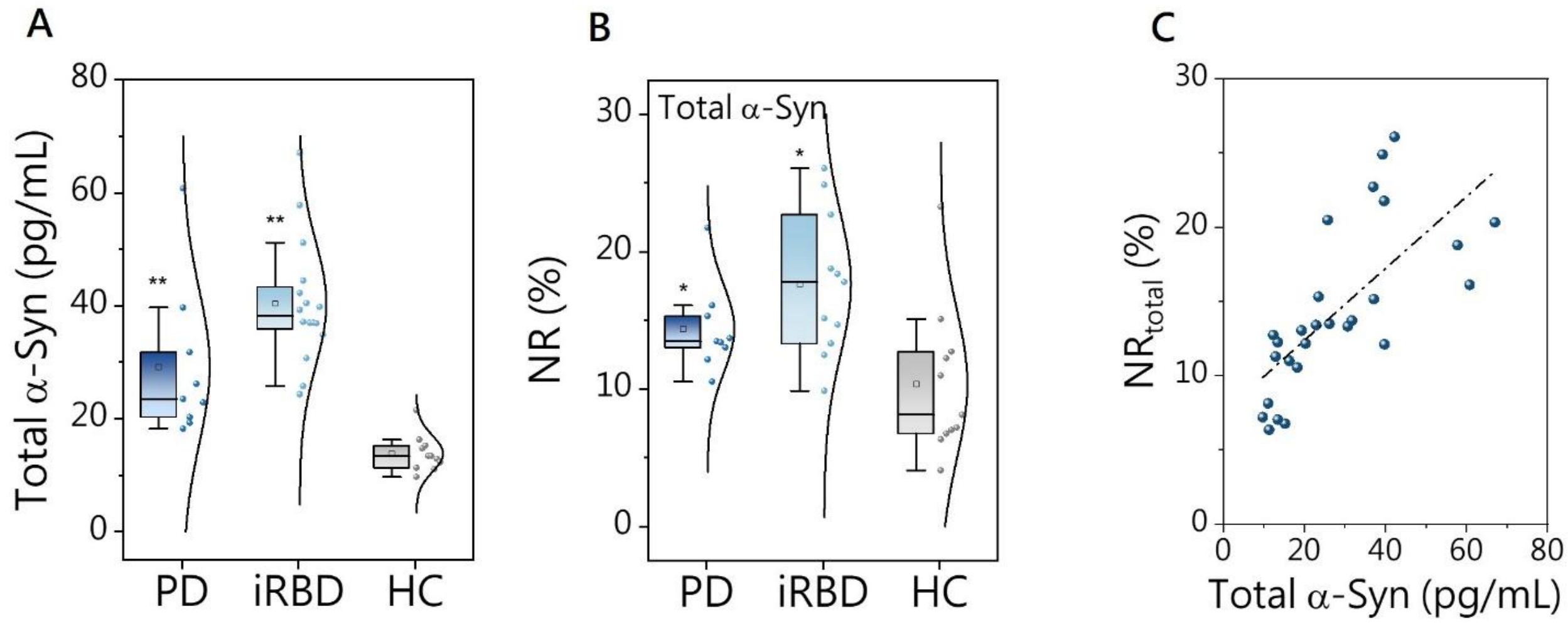

Fig. S22. Comparison between clinical and OECT sensor detection of total α-Syn biomarker. **(A)** MSD measurement showing the concentration of Total α-Syn distributions in different samples. **(B)** OECT sensor output showing the normalized responses of Total α-Syn sensors to all samples tested. **(C)** Comparison between MSD measurement results (x-axis) and OECT measurement results (y-axis). $R^2 = 0.53$. NRs are calculated at $V_{ds} = V_{gs} = -0.5$ V.

Movie S1. OECT-based detection of commercial α-synuclein using a functionalized gate electrode array. This video demonstrates the operational workflow of the OECT sensing assay detecting commercially available total α-synuclein (α-Syn) protein. The procedure includes incubation of a functionalized gate electrode with the blank and target solution, followed by rinsing and subsequent transistor measurements. The resulting changes in the OECT transfer characteristics before and after target exposure illustrate how protein binding at the gate/electrolyte interface modulates the channel current.

REFERENCES

1. C. Jiang, F. Hopfner, D. Berg, M. T. Hu, A. Pilotto, B. Borroni, J. J. Davis, G. K. Tofaris, Validation of α-synuclein in L1CAM-immunocaptured exosomes as a biomarker for the stratification of parkinsonian syndromes. *Mov. Disord.* **36**, 2663–2669 (2021).

2. H. R. Morris, M. G. Spillantini, C. M. Sue, C. H. Williams-Gray, The pathogenesis of Parkinson's disease. *Lancet* **403**, 293–304 (2024).

3. S. Heinzel, D. Berg, T. Gasser, H. Chen, C. Yao, R. B. Postuma, MDS Task Force on the Definition of Parkinson's Disease, Update of the MDS research criteria for prodromal Parkinson's disease. *Mov. Disord.* **34**, 1464–1470 (2019).

4. M. J. Armstrong, M. S. Okun, Diagnosis and treatment of parkinson disease. *JAMA* **323**, 548–560 (2020).

5. E. Tolosa, A. Garrido, S. W. Scholz, W. Poewe, Challenges in the diagnosis of Parkinson's disease. *Lancet Neurol.* **20**, 385–397 (2021).

6. E. Tolosa, G. Wenning, W. Poewe, The diagnosis of Parkinson's disease. *Lancet Neurol.* **5**, 75–86 (2006).

7. N. Vijiaratnam, T. Simuni, O. Bandmann, H. R. Morris, T. Foltynie, Progress towards therapies for disease modification in Parkinson's disease. *Lancet Neurol.* **20**, 559–572 (2021).

8. B. Mollenhauer, C. A. F. von Arnim, Toward preventing Parkinson's disease. *Science* **377**, 818–819 (2022).

9. A. Siderowf, L. Concha-Marambio, D.-E. Lafontant, C. M. Farris, Y. Ma, P. A. Urenia, H. Nguyen, R. N. Alcalay, L. M. Chahine, T. Foroud, D. Galasko, K. Kieburtz, K. Merchant, B. Mollenhauer, K. L. Poston, J. Seibyl, T. Simuni, C. M. Tanner, D. Weintraub, A. Videnovic, S. H. Choi, R. Kurth, C. Caspell-Garcia, C. S. Coffey, M. Frasier, L. M. A. Oliveira, S. J. Hutten, T. Sherer, K. Marek, C. Soto, Parkinson's Progression Markers Initiative, Assessment of heterogeneity among participants in the Parkinson's Progression Markers Initiative cohort

using α-synuclein seed amplification: A cross-sectional study. *Lancet Neurol.* **22**, 407–417 (2023).

10. S. Yan, C. Jiang, A. Janzen, T. R. Barber, A. Seger, M. Sommerauer, J. J. Davis, K. Marek, M. T. Hu, W. H. Oertel, G. K. Tofaris, Neuronally derived extracellular vesicle α-synuclein as a serum biomarker for individuals at risk of developing parkinson disease. *JAMA Neurol.* **81**, 59–68 (2024).

11. S. Yan, W. Zhang, X. Li, S. Dutta, A. R. Castle, Y. Liu, A. Sahoo, C. L. Lam, N. J. F. Gatford, M. T. Hu, C.-z. Li, C. Jiang, B. Shu, G. K. Tofaris, Single extracellular vesicle detection assay identifies membrane-associated α-synuclein as an early-stage biomarker in Parkinson's disease. *Cell Rep. Med.* **6**, 101999 (2025).

12. J. Hällqvist, M. Bartl, M. Dakna, S. Schade, P. Garagnani, M.-G. Bacalini, C. Pirazzini, K. Bhatia, S. Schreglmann, M. Xylaki, S. Weber, M. Ernst, M.-L. Muntean, F. Sixel-Döring, C. Franceschi, I. Doykov, J. Śpiewak, H. Vinette, C. Trenkwalder, W. E. Heywood, K. Mills, B. Mollenhauer, Plasma proteomics identify biomarkers predicting Parkinson's disease up to 7 years before symptom onset. *Nat. Commun.* **15**, 4759 (2024).

13. Y. Zhou, S. Yan, W. Dong, C. Wu, Z. Zhao, R. Wang, Y. Duo, Y. Huang, D. Xu, C. Jiang, Biosensing strategies for amyloid-like protein aggregates. *BMEMat* **2**, e12053 (2024).

14. M. Shi, C. P. Zabetian, A. M. Hancock, C. Ginghina, Z. Hong, D. Yearout, K. A. Chung, J. F. Quinn, E. R. Peskind, D. Galasko, J. Jankovic, J. B. Leverenz, J. Zhang, Significance and confounders of peripheral DJ-1 and α-synuclein in Parkinson's disease. *Neurosci. Lett.* **480**, 78–82 (2010).

15. C. Jiang, F. Hopfner, A. Katsikoudi, R. Hein, C. Catli, S. Evetts, Y. Huang, H. Wang, J. W. Ryder, G. Kuhlenbaeumer, G. Deuschl, A. Padovani, D. Berg, B. Borroni, M. T. Hu, J. J. Davis, G. K. Tofaris, Serum neuronal exosomes predict and differentiate Parkinson's disease from atypical parkinsonism. *J. Neurol. Neurosurg. Psychiatry* **91**, 720–729 (2020).

16. M. Lai, K. Guo, Y. Huang, D. Wang, Y. Duo, J. Yuan, B. Shu, The L1CAM-positive extracellular vesicle-based biomarker as a promising predictor of Parkinson's disease. *Brain-X* **2**, e66 (2024).

17. C. J. Nogueras-Ortiz, E. Eren, P. Yao, E. Calzada, C. Dunn, O. Volpert, F. Delgado-Peraza, M. Mustapic, A. Lyashkov, F. J. Rubio, M. Vreones, L. Cheng, Y. You, A. F. Hill, T. Ikezu, E. Eitan, E. J. Goetzl, D. Kapogiannis, Single-extracellular vesicle (EV) analyses validate the use of L1 cell adhesion molecule (L1CAM) as a reliable biomarker of neuron-derived EVs. *J. Extracell. Vesicles* **13**, e12459 (2024).

18. J. Rivnay, S. Inal, A. Salleo, R. M. Owens, M. Berggren, G. G. Malliaras, Organic electrochemical transistors. *Nat. Rev. Mater.* **3**, 17086 (2018).

19. M. Braendlein, T. Lonjaret, P. Leleux, J. M. Badier, G. G. Malliaras, Voltage amplifier based on organic electrochemical transistor. *Adv. Sci.* **4**, 1600247 (2017).

20. A. Koklu, S. Wustoni, V.-E. Musteata, D. Ohayon, M. Moser, I. McCulloch, S. P. Nunes, S. Inal, Microfluidic integrated organic electrochemical transistor with a nanoporous membrane for amyloid-β detection. *ACS Nano* **15**, 8130–8141 (2021).

21. J. Zheng, X. Jiang, S. Bai, M. Lai, J. Yu, M. Chen, R. Zhou, Y. Jia, H. Yan, Z. Liang, D. Wang, C. Wu, S. Liu, C. Li, J. Yang, Y. Luo, C. Jiang, K. Guo, Clinically accurate diagnosis of Alzheimer's disease via single-molecule bioelectronic label-free profiling of multiple blood extracellular vesicle biomarkers. *Adv. Mater.* **37**, e2505262 (2025).

22. K. Guo, S. Wustoni, A. Koklu, E. Díaz-Galicia, M. Moser, A. Hama, A. A. Alqahtani, A. N. Ahmad, F. S. Alhamlan, M. Shuaib, A. Pain, I. McCulloch, S. T. Arold, R. Grünberg, S. Inal, Rapid single-molecule detection of COVID-19 and MERS antigens via nanobody-functionalized organic electrochemical transistors. *Nat. Biomed. Eng.* **5**, 666–677 (2021).

23. A. Koklu, S. Wustoni, K. Guo, R. Silva, L. Salvigni, A. Hama, E. Diaz-Galicia, M. Moser, A. Marks, I. McCulloch, R. Grünberg, S. T. Arold, S. Inal, Convection driven ultrarapid protein detection via nanobody-functionalized organic electrochemical transistors. *Adv. Mater.* **34**, e2202972 (2022).

24. P. Youssef, W. S. Kim, G. M. Halliday, S. J. G. Lewis, N. Dzamko, Comparison of different platform immunoassays for the measurement of plasma α-synuclein in Parkinson's disease patients. *J. Parkinsons Dis.* **11**, 1761–1772 (2021).

25. M. Norman, T. Gilboa, D. R. Walt, High-sensitivity single molecule array assays for pathological isoforms in Parkinson's disease. *Clin. Chem.* **68**, 431–440 (2022).

26. K. L. Prime, G. M. Whitesides, Self-assembled organic monolayers: Model systems for studying adsorption of proteins at surfaces. *Science* **252**, 1164–1167 (1991).

27. J. C. Love, L. A. Estroff, J. K. Kriebel, R. G. Nuzzo, G. M. Whitesides, Self-assembled monolayers of thiolates on metals as a form of nanotechnology. *Chem. Rev.* **105**, 1103–1170 (2005).

28. N. Hui, X. Sun, S. Niu, X. Luo, PEGylated polyaniline nanofibers: Antifouling and conducting biomaterial for electrochemical DNA sensing. *ACS Appl. Mater. Interfaces* **9**, 2914–2923 (2017).

29. B.-Y. Chang, Conversion of a constant phase element to an equivalent capacitor. *J. Electrochem. Sci. Technol* **11**, 318–321 (2020).

30. W. Tao, P. Lin, J. Hu, S. Ke, J. Song, X. Zeng, A sensitive DNA sensor based on an organic electrochemical transistor using a peptide nucleic acid-modified nanoporous gold gate electrode. *RSC Advances* **7**, 52118–52124 (2017).

31. V. Druet, P. D. Nayak, A. Koklu, D. Ohayon, A. Hama, X. Chen, M. Moser, I. McCulloch, S. Inal, Operation mechanism of n-type organic electronic metabolite sensors. *Adv. Electron. Mater.* **8**, 2200065 (2022).

32. L. Salvigni, P. D. Nayak, A. Koklu, D. Arcangeli, J. Uribe, A. Hama, R. Silva, T. C. Hidalgo Castillo, S. Griggs, A. Marks, I. McCulloch, S. Inal, Reconfiguration of organic electrochemical transistors for high-accuracy potentiometric sensing. *Nat. Commun.* **15**, 6499 (2024).

33. D. A. Bernards, D. J. Macaya, M. Nikolou, J. A. DeFranco, S. Takamatsu, G. G. Malliaras, Enzymatic sensing with organic electrochemical transistors. *J. Mater. Chem.* **18**, 116–120 (2008).

34. J. Nissa, P. Janson, M. Berggren, D. T. Simon, The role of relative capacitances in impedance sensing with organic electrochemical transistors. *Adv. Electron. Mater.* **7**, 2001173 (2021).

35. M. Sharafeldin, S. Yan, C. Jiang, G. K. Tofaris, J. J. Davis, Alternating magnetic field-promoted nanoparticle mixing: The on-chip immunocapture of serum neuronal exosomes for Parkinson's disease diagnostics. *Anal. Chem.* **95**, 7906–7913 (2023).

36. C. Jiang, Y. Fu, G. Liu, B. Shu, J. Davis, G. K. Tofaris, Multiplexed profiling of extracellular vesicles for biomarker development. *Nanomicro Lett.* **14**, 3 (2022).

37. Y. Fu, C. Jiang, G. K. Tofaris, J. J. Davis, Facile impedimetric analysis of neuronal exosome markers in Parkinson's disease diagnostics. *Anal. Chem.* **92**, 13647–13651 (2020).

38. S. Mo, X. Shao, Y. Chen, Z. Cheng, Increasing entropy for colloidal stabilization. *Sci. rep.* **6**, 36836 (2016).

39. S. Kamble, S. Agrawal, S. Cherumukkil, V. Sharma, R. V. Jasra, P. Munshi, Revisiting zeta potential, the key feature of interfacial phenomena, with applications and recent advancements. *ChemistrySelect* **7**, e202103084 (2022).

40. R. Ohno, H. Ohnuki, H. Wang, T. Yokoyama, H. Endo, D. Tsuya, M. Izumi, Electrochemical impedance spectroscopy biosensor with interdigitated electrode for detection of human immunoglobulin A. *Biosens. Bioelectron.* **40**, 422–426 (2013).

41. A. C. Lazanas, M. I. Prodromidis, Electrochemical impedance spectroscopy—A tutorial. *ACS Meas. Sci. Au* **3**, 162–193 (2023).

42. L. C. T. Shoute, G. N. Abdelrasoul, Y. Ma, P. A. Duarte, C. Edwards, R. Zhuo, J. Zeng, Y. Feng, C. L. Charlton, J. N. Kanji, S. Babiuk, J. Chen, Label-free impedimetric immunosensor for point-of-care detection of COVID-19 antibodies. *Microsyst. Nanoeng.* **9**, 3 (2023).
